# Microglial Expression of the Wnt Signaling Modulator *DKK2* Differs between Human Alzheimer’s Disease Brains and Mouse Neurodegeneration Models

**DOI:** 10.1523/ENEURO.0306-22.2022

**Published:** 2023-01-09

**Authors:** Nozie D. Aghaizu, Sarah Jolly, Satinder K. Samra, Bernadett Kalmar, Katleen Craessaerts, Linda Greensmith, Patricia C. Salinas, Bart De Strooper, Paul J. Whiting

**Affiliations:** 1 United Kingdom Dementia Research Institute at University College London, London WC1E 6BT, United Kingdom; 2 Alzheimer's Research UK Drug Discovery Institute (DDI), University College London, London WC1E 6BT, United Kingdom; 3 Department of Neuromuscular Diseases, University College London Queen Square Motor Neuron Disease Centre, Queen Square Institute of Neurology, Queen Square, London WC1N 3BG, United Kingdom; 4 Department of Cell and Developmental Biology, University College London, London WC1E 6BT, United Kingdom; 5 Vlaams Instituut voor Biotechnologie Centre for Brain Disease Research, Leuven 3000, Belgium; 6 Department of Neurosciences and Leuven Brain Institute, Katholieke Universiteit Leuven, Leuven 3000, Belgium

**Keywords:** Alzheimer’s disease, microglia, neurodegeneration, neuroinflammation, Wnt signaling

## Abstract

Wnt signaling is crucial for synapse and cognitive function. Indeed, deficient Wnt signaling is causally related to increased expression of *DKK1,* an endogenous negative Wnt regulator, and synapse loss, both of which likely contribute to cognitive decline in Alzheimer’s disease (AD). Increasingly, AD research efforts have probed the neuroinflammatory role of microglia, the resident immune cells of the CNS, which have furthermore been shown to be modulated by Wnt signaling. The *DKK1* homolog *DKK2* has been previously identified as an activated response and/or disease-associated microglia (DAM/ARM) gene in a mouse model of AD. Here, we performed a detailed analysis of *DKK2* in mouse models of neurodegeneration, and in human AD brain. In *APP/PS1* and *APP^NL-G-F^* AD mouse model brains as well as in *SOD1^G93A^* ALS mouse model spinal cords, but not in control littermates, we demonstrated significant microgliosis and microglial *Dkk2* mRNA upregulation in a disease-stage-dependent manner. In the AD models, these DAM/ARM *Dkk2^+^* microglia preferentially accumulated close to βAmyloid plaques. Furthermore, recombinant DKK2 treatment of rat hippocampal primary neurons blocked WNT7a-induced dendritic spine and synapse formation, indicative of an anti-synaptic effect similar to that of DKK1. In stark contrast, no such microglial *DKK2* upregulation was detected in the postmortem human frontal cortex from individuals diagnosed with AD or pathologic aging. In summary, the difference in microglial expression of the DAM/ARM gene *DKK2* between mouse models and human AD brain highlights the increasingly recognized limitations of using mouse models to recapitulate facets of human neurodegenerative disease.

## Significance Statement

The endogenous negative Wnt regulator *Dkk2* is significantly upregulated at the mRNA level in microglia of Alzheimer’s disease (AD) mouse models, implying that microglia derived Dkk2 protein may detrimentally contribute to a reduced Wnt signaling tone in the AD brain, a known pathophysiological manifestation. Indeed, recombinant DKK2 prevented Wnt-dependent synapse formation in cultured neurons. However, *DKK2* upregulation was not recapitulated in postmortem human AD brains. The success of neurodegeneration animal models has relied on pathophysiology that for the most part correctly modelled human disease. Increasingly, however, limitations to the validity of mouse models to recapitulate human neurodegenerative disease have become apparent, as evidenced by the present study by the difference in microglial *DKK2* expression between AD mouse models and human AD brain.

## Introduction

Microglia, the resident immune cells of the CNS, contribute both beneficially and detrimentally to Alzheimer’s disease (AD) in a context-dependent manner, thus rendering their response to AD heterogeneous in nature. So too is their phenotype at the transcriptomic, proteomic, epigenomic, metabolomic, and morphologic level leading to the identification of spatiotemporally distinct microglial subpopulations (for review, see Masuda et al., 2020; Paolicelli et al., 2022). Disease-associated (DAM) or activated response microglia (ARM; henceforth: DAM/ARM) represent a subpopulation associated with the neurodegenerative brain (Keren-Shaul et al., 2017; Sala Frigerio et al., 2019). Transitioning from homeostatic to DAM/ARM-state requires TREM2 (triggering receptor expressed on myeloid cells-2; Keren-Shaul et al., 2017). A bona fide receptor for βAmyloid, TREM2 ligation activates microglia and orchestrates a gene regulatory response that increases inflammatory signaling, phagocytosis, and proliferation, a response thought to restrict development of AD (for review, see Gratuze et al., 2018).

TREM2 regulates microglial proliferation and survival by activating, among others, the canonical Wnt/β-catenin pathway (Zheng et al., 2017; for review, see Aghaizu et al., 2020). Indeed, several genes upregulated by TREM2 in response to AD pathology are related to proliferation and Wnt signaling (Meilandt et al., 2020). The canonical Wnt signaling modulatory gene *Dkk2* (Mao and Niehrs, 2003) was upregulated downstream of *Trem2* in DAM/ARM cells in *APP/PS1*, *PS2APP*, *5xFAD*, and *APP^NL-G-F^* AD mouse models in separate studies, making it a putative DAM/ARM marker gene (Friedman et al., 2018; Sala Frigerio et al., 2019; Meilandt et al., 2020). Database searches further indicate that while control CNS *DKK2/Dkk2* expression levels are generally low across the various cell types, they are, respectively, either similar or greater in nonmicroglial CNS cell populations compared with microglia in human and mouse single-cell RNA sequencing (RNA-Seq) studies (Zhang et al., 2014, 2016; Friedman et al., 2018). The secreted protein DKK2 belongs to the Dickkopf family of Wnt modulators (Niehrs, 2006). Its homolog DKK1 antagonizes Wnt signaling through Frizzled Wnt receptors by sequestering the co-receptor LRP5/6 (Bafico et al., 2001; Mao et al., 2001). The reduced Wnt signaling tone evident in AD is at least partially because of Aβ fibril-induced upregulation of *DKK1/Dkk1* in human AD and AD mouse models (Caricasole et al., 2004; Rosi et al., 2010; Killick et al., 2014; Sellers et al., 2018; Jackson et al., 2019). This was synaptotoxic in *in vitro* and *in vivo* models (Purro et al., 2012; Galli et al., 2014; Marzo et al., 2016; Elliott et al., 2018; Sellers et al., 2018), and potentially also in human AD (Jackson et al., 2019).

Much less is known about the role of microglial *DKK2* in the CNS, not to mention in AD. In cell lines, DKK2 can both antagonize and agonize Wnt-LRP6 signaling depending respectively on the presence or absence of the second co-receptor Kremen2 (Mao and Niehrs, 2003). During neural crest specification, DKK2 agonizes Wnt signaling (Devotta et al., 2018). Conversely, in cancer studies DKK2 generally inhibits Wnt signaling (Kuphal et al., 2006; Sato et al., 2007; Maehata et al., 2008; Hirata et al., 2009; Zhu et al., 2012; Mu et al., 2017). Furthermore, cancer cell-secreted DKK2 suppresses immune cell activation via an unconventional Wnt-unrelated pathway (Xiao et al., 2018). In the aforementioned single-cell and bulk cell gene expression studies on neurodegeneration mouse models, *Dkk2* was upregulated in microglia, but no information on the spatial relationship between *Dkk2^+^* microglia and neurodegenerative pathology or the biological role of this upregulation was provided (Friedman et al., 2018; Sala Frigerio et al., 2019; Meilandt et al., 2020). To address this gap in our knowledge, we performed a histologic assessment of microglial *Dkk2/DKK2* upregulation in several mouse models and in human AD and furthermore investigated the effect of recombinant DKK2 on cultured primary neurons.

Here, we report significant microgliosis and microglial *Dkk2* mRNA upregulation in a disease-stage-dependent manner in *APP/PS1*, and *APP^NL-G-F^* AD mouse model brains. Clustering of *Dkk2*^+^ microglia around amyloid plaques was often more pronounced than that of *Dkk2^–^* microglia. In cultured rat neurons, recombinant DKK2 blocked Wnt-dependent synapse formation. Crucially however, microglial *DKK2* upregulation was not detected in postmortem human brain from individuals diagnosed with AD or pathologic aging. This nonuniversality of what was a putative DAM/ARM marker gene highlights the increasingly recognized limitations of using animal models to recapitulate facets of human neurodegenerative disease.

## Materials and Methods

### Mice

Mouse CNS tissue was obtained from the following sources: brain tissue from male and female *B6.Cg-Tg*(*APPswe, PSEN1dE9*) mice (Jankowsky et al., 2004; RRID:MMRRC_034829-JAX; abbreviated *APP/PS1*) and age-matched wild-type *C57BL/6J* control mice at 3, 8, and 12 months was purchased from WuXi AppTec. Brain tissue from male homozygous *B6.129S5-Apptm3.1Tcs* mice (Saito et al., 2014; RRID:IMSR_RBRC06344; backcrossed for at least two generations with *C57BL/6J* mice; here referred to as *APP^NL-G-F^*) and age-matched wild-type *C57BL/6J* control mice at 7 and 24 months was kindly provided by the De Strooper lab. Spinal cord tissue from female mice expressing mutant human *SOD1^G93A^* (*B6SJL*-*Tg[SOD1^G93A^]1Gur/J*; Gurney et al., 1994; RRID:IMSR_JAX:002726; abbreviated *SOD1^G93A^*) and age-matched female control mice expressing wild-type human *SOD1* (*B6SJLTg[SOD1]2Gur/J*; Gurney et al., 1994; RRID:IMSR_JAX:002297; abbreviated *SOD1^WT^*) at 50, 100, and 120 d was kindly provided by the Greensmith lab. Colonies were maintained by breeding male heterozygous carriers with female (*C57BL/6 × SJL*) F1 hybrids. Mice were genotyped for the human *SOD1* transgene from ear or tail genomic DNA.

In every case, mice were housed according to the appropriate institution’s ethical requirements, and in compliance to the country’s laws for animal research. Typically, mice were housed in standard individually ventilated cages with less than or equal to three mice per cage at 21 ± 1°C with relative humidity 55 ± 10% and maintained on a 12/12 h light/dark cycle with access to food (standard pellets), water, and nesting material provided *ad libitum* via an overhead rack. At the onset of pathology, affected animals were provided with food pellets soaked in water at ground level to ensure sufficient nourishment and hydration. Cages were checked daily to ensure animal welfare. Body weight was assessed regularly to ensure no weight loss. For animals housed at WuXi AppTec, studies were reviewed and approved by Institutional Animal Care and Use Committee (IACUC) of WuXi AppTec (Suzhou) Co, Ltd. For animals housed at VIB/kU Leuven, studies were approved by the kU Leuven Ethical Committee and in accordance with European Directive 2010/63/EU. For animals housed at UCL, studies were conducted following the guidelines of the UCL Institute of Neurology Genetic Manipulation and Ethic Committees and in accordance with the European Community Council Directive of November 24, 1986 (86/609/EEC). Animal experiments were undertaken under license from the United Kingdom Home Office in accordance with the Animals (Scientific Procedures) Act 1986 (Amended Regulations 2012) and were approved by the Ethical Review Panel of the Institute of Neurology.

For tissue collection, animals were injected with terminal anesthesia (pentobarbital sodium, Euthatal) and were transcardially perfused with PBS by trained personnel.

### Rats

Animal experiments were undertaken under license from the United Kingdom Home Office in accordance with the Animals (Scientific Procedures) Act 1986 (Amended Regulations 2012) and in compliance with the ethical standards at University College London (UCL). Timed matings were set up for Sprague Dawley rats for subsequent harvesting of embryos at embryonic day (E)18. Pregnant rat dams were killed using Isoflurane and cervical dislocation.

### Human postmortem tissue

Anonymized human samples from control, pathologic aging, and AD subjects were obtained from the Queen’s Square Brain Bank for Neurologic Disorders (QSBB) and NeuroResource, UCL Institute of Neurology, University College London. All samples were obtained with informed consent in accordance with the Human Tissue Act 2004 and under the UCL Institute of Neurology HTA material transfer agreement UCLMTA1/17 approved by the NHS Research Ethics Committee. Postmortem frontal cortex biopsy tissue was harvested, snap-frozen, and stored at −80°C until further tissue processing. All experiments were performed in accordance with relevant guidelines and regulations. Sample information including demographic data, disease classifications and postmortem intervals is shown in Extended Data Table 6-1.

### Tissue processing

Mouse brain tissue, freshly harvested on transcardial perfusion with PBS, was postfixed by immersion in 4% paraformaldehyde (PFA) in PBS overnight at 4°C followed by overnight immersion in and equilibration to 20% sucrose in PBS at 4°C. Mouse brains were split into three segments by applying 2 equidistant coronal slice cuts along the rostro-caudal axis, resulting in an olfactory bulb containing rostral-most segment, a hippocampus containing middle segment and the caudal-most cerebellar segment. After embedding in OCT (CellPath) and freezing in 2-methylbutane (Sigma) prechilled in liquid Nitrogen, the middle segment was coronally cryosectioned at 15-μm thickness on a Leica CM1860UV cryostat (Leica) and sections containing clearly defined hippocampus were transferred onto Superfrost Plus Gold microscopy slides (ThermoScientific).

Mouse spinal cord tissue was processed identically but split only into two segments by applying a transverse slice cut rostral to the lumbar enlargement, resulting in a rostral cervical/thoracic segment and a caudal lumbar segment. The cryo-embedded lumbar segment was transversally cryosectioned at 15-μm thickness and sections containing clearly defined L5 lumbar spinal cord were transferred onto Superfrost Plus Gold microscopy slides (ThermoScientific).

Human frontal cortex brain tissue was cryosectioned at 15-μm thickness, sections were transferred onto Superfrost Plus Gold microscopy slides (ThermoScientific) and dried for 10 min at 40°C.

Human and mouse sections were stored at −80°C until staining.

### mRNA fluorescence *in situ* hybridization (FISH)

mRNA fluorescence *in situ* hybridization for mouse *Dkk2* and human *DKK2*, *TREM2*, and *P2RY12* mRNA was performed on mouse brain/spinal cord and human frontal cortex cryosections respectively, by using the Multiplex Fluorescent V2 Assay kit (ACD Bio).

Briefly, mouse cryosections were thawed and dried at 40°C for 4 min before postfixation with 4% PFA in PBS at room temperature (RT) for 10 min. OCT residue was washed off by applying 1× PBS for 5 min at RT. Sections were treated with RNAScope H_2_O_2_ for 4 min at RT and subsequently washed 2× 3 min with UltraPure Distilled Water (Invitrogen) at RT. Microscopy slides containing cryosections were submerged for 4 min in boiling 1× RNAScope target retrieval solution followed by immediate submersion in UltraPure Distilled Water. Cryosections were dehydrated in 100% ethanol at RT for 2 min and allowed to air dry at RT for 5 min. Cryosections were subsequently treated with RNAScope Protease IV at RT for 15 min and washed 2× 3 min at RT with 1× PBS. RNAScope probes were allowed to hybridize to cryosections for 2 h at 40°C (*Mm*-*Dkk2*-C1, 404841; *Mm-Ppib*-C1 (positive control probe), 313911; *E. coli*-*Dapb*-C1 (negative control probe), 310043). Probes were detected with TSA-Cy3 (PerkinElmer, FP1170) using the RNAScope branched DNA amplification principle as per the manufacturer’s instructions. Subsequently, cryosections were further immunohistochemically processed (see below).

Human cryosections were processed similarly as previously described (Jolly et al., 2019). Briefly, cryosections were thawed and dried at 40°C for 4 min before postfixation with chilled 4% PFA in PBS at 4°C for 30 min followed by 2× 2-min washes with 1× PBS at RT. Cryosections were then dehydrated in an ethanol dilution series (50%, 70%, 2× 100%) at RT for 5 min each and subsequently allowed to air dry at RT for 5 min. Sections were treated with RNAScope H_2_O_2_ for 10 min at RT and subsequently washed 2× 2 min with 1× PBS. Microscopy slides containing cryosections were submerged for 10 min in boiling 1× RNAScope target retrieval solution followed by 2× 2-min washes with 1× PBS. Cryosections were subsequently treated with RNAScope Protease IV at RT for 20 min and washed 2× 3 min at RT with 1× PBS. RNAScope probes were allowed to hybridize to cryosections for 2 h at 40°C [*Hs*-*TREM2*-C1, 420491; *Hs*-*DKK2*-C2, 531131-C2; *Hs*-*P2RY12*-C3; 450391-C3; *Hs*-*PPIB*-C1 (positive control probe), 313901; *E. coli*-*Dapb*-C1 (negative control probe), 310043]. C2 and C3 probes were diluted in C1 probe solution at a 1:50 ratio. Probes were detected with TSA-Cy3 (PerkinElmer, FP1170), Opal 620 (Akoya, FP1495001KT), and TSA-Cy5 (PerkinElmer, REF FP1168) using the RNAScope branched DNA amplification principle as per the manufacturer’s instructions. Subsequently, cryosections were further immunohistochemically processed (see below).

### Primary hippocampal neuron cultures

Primary rat hippocampal neuron cultures were prepared from embryonic day 18 (E18) Sprague Dawley rat embryos. One day before neuron isolation, eight well chamber slide dishes (Miltenyi Biotec) were coated over night with 1 mg/ml poly-L-lysine in borate buffer (boric acid, 3.1 g/l; borax 4.8 g/l; pH 8.5). On the day of the neuron isolation, dishes were washed 3× 20 min with UltraPure Distilled Water, filled with plating medium [Neurobasal (ThermoFisher) supplemented with 1× B27 (ThermoFisher), 1× GlutaMAX (ThermoFisher), 1× penicillin-streptomycin (ThermoFisher), 25 μm L-glutamate (Sigma)], and preequilibrated at 5% CO_2_, 37°C. Hippocampi were dissected from brain tissue using sterilized tools (Dumont #5 fine tip tweezers, Dumont #7 curved forceps, Student Vannas Scissors 9 cm long/straight; Fisherbrand) and collected in ice cold HBSS (Invitrogen). Following three washes with fresh ice cold HBSS, hippocampi were enzymatically dissociated by incubation in accutase (ThermoFisher) at 37°C for 10 min, providing manual agitation every 2–3 min. Hippocampi were then washed three times with prewarmed (37°C) HBSS, followed by mechanical dissociation into a single-cell suspension by trituration in HBSS using a 1-ml pipette. Live cell density was determined using the Countess 3 automated cell counter (ThermoFisher) and cells were plated onto eight-well chamber slides at a density of 43,000 cells/cm^2^ and cultured in an incubator at 37°C/5% CO_2_. Half medium changes were performed twice per week with maintenance medium: Neurobasal, supplemented with 1× B27, 1× GlutaMAX, 1× penicillin-streptomycin.

Neuronal transfection with the DNA construct *pHR hsyn:EGFP* [Keaveney et al., 2018; kind gift from Xue Han (Addgene plasmid #114215; http://n2t.net/addgene:114215; RRID:Addgene_114215)] was performed at 7 d *in vitro* (DIV) using the Neuromag magnetofection method (OzBiosciences). Briefly, for every 40,000 cells plated per well of an eight-well chamber slide dish, 0.5-μg DNA was mixed and complexed with 1-μl Neuromag transfection reagent in 100 μl of OptiMem (all reagents at room temperature). Following 20 min of incubation at room temperature, the transfection mix was added dropwise to neuronal cultures and the culture dish was placed on a magnetic plate (OzBiosciences) preequilibrated to 37°C inside an incubator for the magnetofection step. After 20 min of magnetofection in the incubator, cell culture dish was removed from the magnetic plate and normal cell culture resumed.

Recombinant protein treatment was performed at 21 DIV for 24 h: human DKK2 (Bio-Techne, 6628-DK-010/CF, 100 ng/ml), human DKK1 (Bio-Techne, 5439-DK-010/CF, 100 ng/ml), human WNT7a (Bio-Techne, 3008-WN-010/CF, 200 ng/ml); 100 ng/ml bovine serum albumin (BSA) in 1× PBS heat inactivated at 95°C for 5 min was used as control.

Fixation was performed following 24 h of recombinant protein treatment using 4% PFA/4% sucrose (Sigma) in 1× PBS at RT for 15 min. Neurons were subsequently washed 3× with 1× PBS.

### Immunocytochemistry and immunohistochemistry

Tissue sections stained by mRNA FISH and fixed primary neurons were washed with 1× PBS and blocked in 1× PBS supplemented with 5% (v/v) goat serum (Bio-Rad), 1% (wt/vol) BSA (Sigma) and 0.1% (v/v) Triton X-100 (Sigma) at RT for 1 h. Primary antibodies were diluted in blocking solution and applied to samples at 4°C overnight. Primary antibodies used in this study were: βAmyloid (BioLegend, 803001, RRID:AB_2564653, 1:200), GFAP (Sigma, G3893, RRID:AB_477010, 1:500), Homer (SynapticSystems, 160003, RRID:AB_887730, 1:500), Iba1 (Fujifilm Wako, 019-19741, RRID:AB_839504, 1:250), misfolded SOD1 (Médimabs, MM-0070-P, RRID:AB_10015296, 1:100), vGlut (MerckMillipore, AB5905, RRID:AB_2301751, 1:300); negative controls omitted the primary antibody. This was followed by 4× 10 min washes in 1× PBS at RT and subsequent application of suitable goat Alexa Fluor Plus secondary antibodies (488/546/647) diluted 1:500 in blocking solution at RT for 2 h. Samples were then washed 4× 10 min with 1× PBS at RT. Cryosections only were treated with 1× TrueBlack (Biotium) at RT for 30 s to quench autofluorescence caused by the accumulation of lipofuscin and other protein aggregates, followed by 2× washes with 1× PBS. Nuclei of samples were counterstained with DAPI (Sigma; shown in blue in all confocal images) at 1 μg/ml in PBS and samples were mounted using DAKO Fluorescence Mounting Medium (Agilent).

### Microscopy

Stained tissue was imaged using a Zeiss LSM 880 confocal laser scanning microscope fitted with 40× (NA = 1.3) and 63× (NA = 1.4) objectives and photomultiplier tubes to detect fluorescence emission. For image acquisition, *xyz* confocal stacks were captured at a resolution of 1024 × 1024 pixels and at a step size of 1 μm. Microscope settings were established during first acquisition and subsequently not further modified. Four distinct fields of view (FOVs) were imaged from two representative sections per sample.

For image acquisition of transfected primary rat hippocampal neurons following recombinant protein treatment, whole neurons were acquired using the 40× objective and secondary dendrites were acquired with higher magnification using the 63× objective with an additional 3.5× zoom at a resolution of 1024 × 1024 pixels and at a step size of 0.5 μm. A total of 15 neurons and accompanying secondary dendrites spread across three biological repeats were imaged per condition.

### Image processing and analysis

All images acquired from mouse tissue were processed and analyzed in Fiji/ImageJ (Schindelin et al., 2012). *xyz* confocal stacks were collapsed into maximum *z* projections. Microgliosis was assessed by measuring both the number of microglia (DAPI^+^ nuclei embedded within typical microglial Iba1 immunoreactivity) and the total 2D surface area of Iba1 immunoreactivity within the acquired field of view. For area quantification, Iba1 immunoreactivity was processed by applying the “Remove Outliers” function to remove nonspecific noise (bright, radius = 2, threshold = 50), followed by thresholding at 35/255 to define the signal range, and two further rounds of the “Remove Outliers” function, to fill-in nuclear and other gaps in Iba1 staining (dark, radius = 1), and to further remove nonspecific noise (bright, radius = 3). The created Iba1 surface area was measured and used as a mask within which the *Dkk2* mRNA FISH signal surface area, thresholded to 30/255, was quantified. Normalized *Dkk2* area per microglial cell was determined by dividing the total measured *Dkk2* area by the number of detected Iba1^+^/DAPI^+^ microglia within a given field of view.

Human frontal cortex image acquisitions were first subjected to “Linear unmixing” with automatic fluorophore detection within the Zeiss Zen Black software (Zeiss) to remove overlapping signals between the five fluorophore channels. Unmixed and maximum z projected images were subsequently processed and analyzed using the HALO FISH-IF v2.0.4 module (Indica Labs). The *DKK2* mRNA FISH signal surface area associated with *TREM2/P2RY12* double positive microglia cells was quantified. To achieve this, cell nuclei and their *xy* coordinates were recorded based on DAPI signal. Probe detection was optimized based on signal size, intensity of positive probe pixels and contrast threshold parameter settings (see Extended Data Table 6-2). The maximum distance threshold for probe signal assignment to nuclei was 25 μm. We classified cells positive for *P2RY12* and *TREM2* as microglia (DAPI^+^/*P2RY12/TREM2*^+^), determined their number, and measured the surface area of *DKK2* mRNA FISH signal associated with such DAPI^+^/*P2RY12/TREM2*^+^ cells. Normalized *DKK2* area per microglial cell was determined by dividing the total measured *DKK2* area by the number of detected DAPI^+^/*P2RY12/TREM2*^+^microglia within a given field of view.

Microglia-βAmyolid plaque distance analysis: we determined the 2D Euclidian distance of microglia to the proximal most βAmyolid plaque dense core in maximum projected images according to the following Fiji/ImageJ methodology: an intensity threshold was applied to the image channel containing βAmyloid immunostaining to identify the plaque dense core, which was usually more intensely labeled compared with the plaque periphery; because of the heterogeneous nature of βAmyloid plaques, threshold values were determined for each acquired image. In early-stage *APP/PS1,* and *APP^NL-G-F^* AD mouse or littermate control tissue devoid of βAmyolid plaques, plaque dense core “placeholders” were randomly placed on confocal images by digitally drawing appropriately dimensioned white ellipses on the color channel assigned to βAmyloid immunostaining using Fiji/ImageJ, followed by intensity threshold application as above. The binary dense core image generated in the previous step was subjected to the “Exact Signed Euclidian Distance Transform (3D)” (EDT) plug-in to create a 2D map where distance to the closest dense core was encoded in gray values from −1024 (furthest possible distance) to 0 (at dense core edge). *xy* position landmarks of DAPI^+^ microglia nuclear centers were placed on a binary image, which in turn was redirected to the EDT image in the “Set Measurements” window, selecting “Mean gray value” as measurement output. Note that *xy* positions of human microglia exported from HALO FISH-IF v2.0.4 module were imported into Fiji/ImageJ using the macro “ImportXYcoordinates.ijm.” Gray values at microglial *xy* positions were obtained using the “Analyze Particles” function and converted into distance units by multiplying the gray value by the image *xy* pixel dimension (0.13495 μm) to yield microglia-βAmyloid plaque distances.

Dendritic spine and synapse analysis on *hSyn:EGFP*-expressing primary rat hippocampal neurons following recombinant protein treatment was performed using IMARIS software. Briefly, the “Filament” tool was used to semi-automatically specify the secondary dendrite within an image file, followed by the detection of dendritic spines by manual identification. Postsynaptic Homer immunoreactivity usually manifested as puncta in dendrites, especially within dendritic spines. To quantify the number of Homer puncta exclusively within the transfected secondary dendrite of interest, the GFP signal was used to create an exclusion mask using the “Surface” tool to isolate the Homer signal within the transfected dendrite. Homer puncta were subsequently identified using the “Spot” detection tool set to a detection diameter of 0.45 μm; background Homer signal was excluded by thresholding using the “Quality” filter. Presynaptic vGlut puncta in the entire image were similarly identified using the “Spot” detection tool at 0.45-μm diameter. Synapses were assumed using the “Colocalize spots” function within the “Spot” detection tool when there was a maximum distance of 1 μm between Homer and vGlut puncta.

### Experimental design and statistical analysis

All means are stated ± SD. For the histologic study aspects, *N* = number of subjects (humans or animals) and *n* = number of fields of view. For qualitative and quantitative histologic assessments, we typically examined at least four subjects per group, imaging at least four different fields of view from two cryosections per subject, which met previously conducted sample size calculations according to Rosner (2015) with data inputs from Friedman et al. (2018). For the cytological study aspects, *N* = number of biological repeats, *n* = number of technical repeats (cells analyzed). We used GraphPad Prism software (GraphPad Software Inc.) for statistical analyses. D’Agostino and Pearson test was used to assess the normality of datasets. For the comparison of one independent variable between more than two groups, we used one-way ANOVA with Tukey’s multiple comparison test. For statistical tests involving two independent variables we used two-way ANOVA with Šidák multiple comparisons test; where data points were missing, Mixed-effects analysis with Šidák multiple comparisons test was utilized. Significance was accepted at *p *≤* *0.05 (see Table 1; alphabetical superscripts in results section and figure legends refer to Table 1).

**Table 1:** Statistical table

	Data structure	Type of test	Power
*^a^*	Normal distribution	Two-way ANOVA with Šidák multiple comparisons test	*p* = 0.0101 (tp), *p* = 0.0002 (gt)
*^b^*	Normal distribution	Two-way ANOVA with Šidák multiple comparisons test	*p* = 0.1821 (tp), *p* = 0.0018 (gt)
*^c^*	Normal distribution	Two-way ANOVA with Šidák multiple comparisons test	*p* = 0.0245 (tp), *p* = 0.0013 (gt)
*^d^*	Normal distribution	Mixed-effects analysis with Šidák multiple comparisons test	*p* = 0.0005 (tp), *p* = 0.0051 (gt)
*^e^*	Normal distribution	Two-way ANOVA with Šidák multiple comparisons test	*p* = 0.0119 (tp), *p* = 0.0288 (gt)
*^f^*	Normal distribution	Two-way ANOVA with Šidák multiple comparisons test	*p* = 0.1363 (tp), *p* = 0.0652 (gt)
*^g^*	Normal distribution	Two-way ANOVA with Šidák multiple comparisons test	*p* = 0.0240 (tp), *p* = 0.0004 (gt)
*^h^*	Normal distribution	Two-way ANOVA with Šidák multiple comparisons test	*p* = 0.0784 (tp), *p* = 0.0093 (gt)
*^i^*	Normal distribution	Two-way ANOVA with Šidák multiple comparisons test	*p* < 0.0001 (tp), *p* < 0.0001 (gt)
*^j^*	Normal distribution	Two-way ANOVA with Šidák multiple comparisons test	*p* = 0.0030 (tp), *p* = 0.0006 (gt)
*^k^*	Normal distribution	Two-way ANOVA with Šidák multiple comparisons test	*p* = 0.0003 (tp), *p* = 0.0391 (gt)
*^l^*	Normal distribution	Two-way ANOVA with Šidák multiple comparisons test	*p* = 0.3563 (tp), *p* = 0.7931 (gt)
*^m^*	Normal distribution	Two-way ANOVA with Šidák multiple comparisons test	*p* = 0.1691 (tp), *p* = 0.7041 (gt)
*^n^*	Normal distribution	One-way ANOVA with Tukey’s multiple comparison test	Various
*^o^*	Normal distribution	One-way ANOVA with Tukey’s multiple comparison test	Various
*^p^*	Normal distribution	One-way ANOVA with Tukey’s multiple comparison test	Various
*^q^*	Normal distribution	Two-way ANOVA with Šidák multiple comparisons test	*p* < 0.0001 (tp), *p* = 0.0001 (gt)
*^r^*	Normal distribution	Two-way ANOVA with Šidák multiple comparisons test	*p* < 0.0001 (tp), *p* = 0.0001 (gt)
*^s^*	Normal distribution	Two-way ANOVA with Šidák multiple comparisons test	*p* = 0.0154 (tp), *p* = 0.0193 (gt)
*^t^*	Normal distribution	Two-way ANOVA with Šidák multiple comparisons test	*p* = 0.9125 (tp), *p* = 0.0073 (gt)
*^u^*	Normal distribution	One-way ANOVA with Tukey’s multiple comparison test	*p* = 0.0023
*^v^*	Normal distribution	One-way ANOVA with Tukey’s multiple comparison test	*p* = 0.0309
*^w^*	Normal distribution	One-way ANOVA with Tukey’s multiple comparison test	*p* = 0.4507
*^x^*	Normal distribution	One-way ANOVA with Tukey’s multiple comparison test	*p* = 0.7689
*^y^*	Normal distribution	One-way ANOVA with Tukey’s multiple comparison test	*p* = 0.8650
*^z^*	Normal distribution	One-way ANOVA with Tukey’s multiple comparison test	*p* = 0.2349
*^ab^*	Normal distribution	One-way ANOVA with Tukey’s multiple comparison test	*p* = 0.1056

### Data, software, and code availability

The datasets generated during and/or analyzed during the current study are available from the corresponding authors on request. The Fiji/ImageJ macro “ImportXYcoordinates.ijm” is available on the Github repository available via https://github.com/DominicAghaizu/ImageJMacros/blob/main/ImportXYcoordinates.ijm.

## Results

### Microgliosis and microglial *Dkk2* upregulation in *APP^NL-G-F^* mice

We first investigated the microglial *Dkk2* expression pattern in the *APP^NL-G-F^* knock-in AD mouse model, which develops robust pathology from the physiological expression of humanized mouse amyloid precursor protein (*App*) harboring Swedish, Beyreuther/Iberian, and Arctic mutations (Saito et al., 2014). To this end, we performed mRNA FISH on coronal brain cryosections to detect *Dkk2* mRNA *in situ* and acquired images from the motor cortex and the stratum pyramidale, with adjacent stratum oriens and stratum radiatum, of the hippocampal CA1 region, which are brain regions burdened by βAmyloid plaques, neurofibrillary tangles and neuronal degeneration in AD patients and animal models. This was paired with immunohistochemical labeling using antibodies against Iba1 and βAmyloid to assess microglial *Dkk2* expression, as suggested previously (Friedman et al., 2018; Sala Frigerio et al., 2019; Meilandt et al., 2020), and to evaluate the spatial relationship between microglia and βAmyloid plaque lesions.

**Figure 1.**
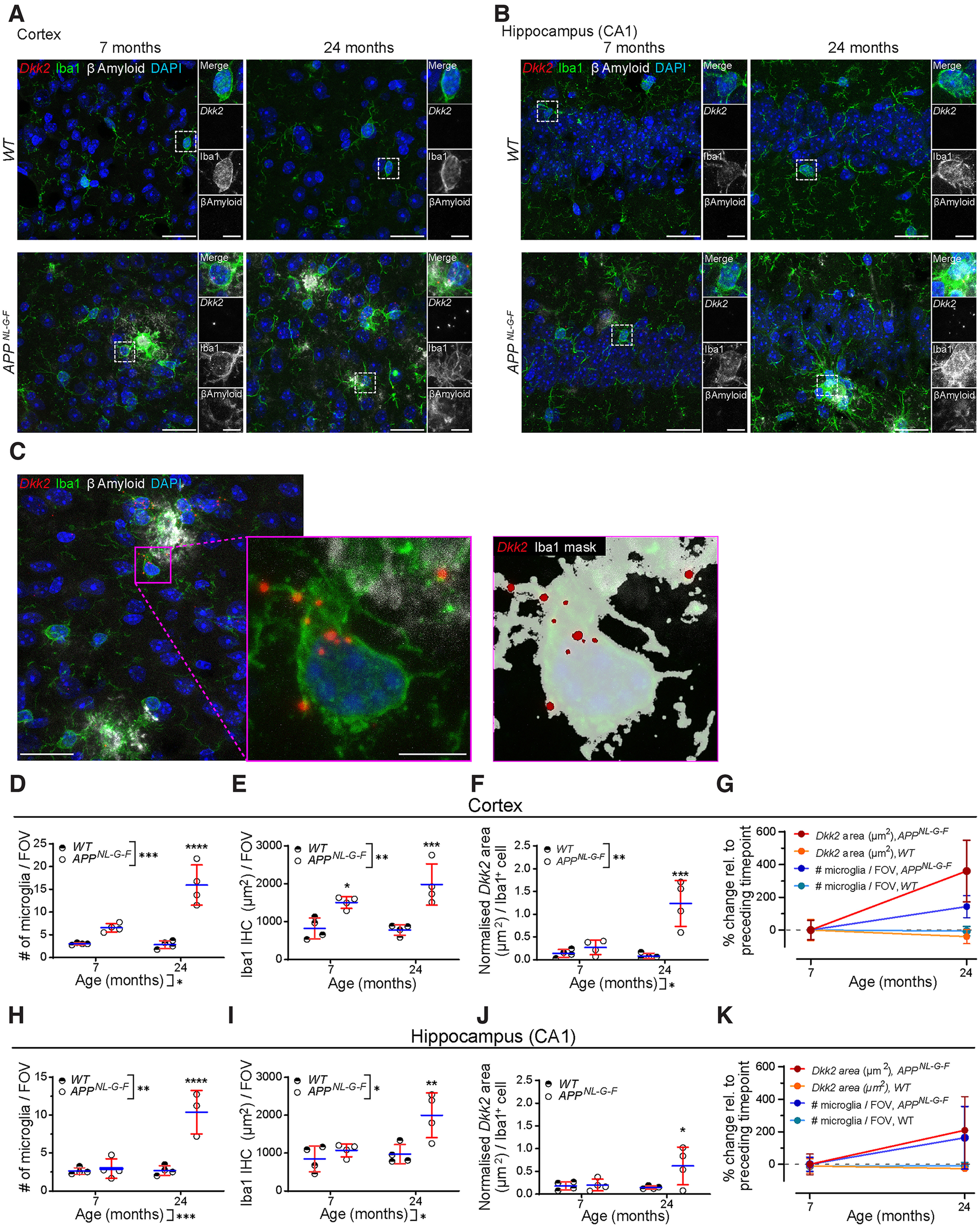
Microgliosis and microglial *Dkk2* upregulation in *APP^NL-G-F^* vs. wild-type (*WT*) mice. *Dkk2* mRNA. FISH as well as microglial Iba1 and βAmyloid IHC labeling in the motor cortex (***A***) and CA1 hippocampus (***B***) of *APP^NL-G-F^* mice. Boxed regions of interest (ROIs) were magnified for increased detail. ***C***, FIJI/ImageJ analysis workflow to quantifying punctated *Dkk2* mRNA FISH signal in Iba1-labeled (microglial) cells. Iba1 staining based analysis mask was generated, within which Dkk2 signal was quantified. ***D–G***, Microgliosis and *Dkk2* expression quantification in the *APP^NL-G-F^* motor cortex. ***D***, Quantification of microglia numbers per maximum projected field of view (FOV; 1.8 × 10^−2^ mm^2^). ***E***, Iba1 IHC surface area per maximum projected FOV. ***F***, Normalized *Dkk2* mRNA FISH signal area per DAPI^+^/Iba^+^ microglial cell. ***G***, Comparative % changes of *Dkk2* expression and microglia numbers during time course. ***H–K***, Microgliosis and *Dkk2* expression quantification in the *APP^NL-G-F^* CA1 hippocampus. ***H***, Quantification of microglia numbers per maximum projected FOV. ***I***, Iba1 IHC surface area per maximum projected FOV. ***J***, Normalized *Dkk2* mRNA FISH signal area per DAPI^+^/Iba^+^ microglial cell. ***K***, Comparative % changes of *Dkk2* expression and microglia numbers during time course. Individual data points represent the average of four FOVs analyzed for each animal (***D–F***, ***H–J***) or total averages from all animals per group (***G***, ***K***). *N* = 4 animals per condition and time point, *n* = 4 different fields of view/animal and brain region. Scale bars: 25 μm (***A–C***) and 5 μm (magnified ROIs). Data show mean +/– SD. Two-way ANOVA with multiple comparisons test. *p < 0.05, **p < 0.01, ***p < 0.001, ****p < 0.0001 (^a–f^). See also Extended Data Figure 1-1.

As expected, the brains of wild-type control littermate mice at 7 or 24 months were devoid of βAmyloid plaques and exhibited normally tiled Iba1^+^ microglia (Fig. 1*A*,*B*). In stark contrast, we detected βAmyloid plaques in the cortex and CA1 of age-matched transgenic *APP^NL-G-F^* mice at 7 and 24 months (Fig. 1*A*,*B*). This was accompanied by robust microgliosis as assessed by both normalized microglia cell count (DAPI^+^/Iba1^+^ cells) and area of Iba1 signal in maximum *z*-projected image stacks (Fig. 1*C*; note that the microglia spatial distribution will be addressed below). In the cortex, the number of microglia was significantly higher in *APP^NL-G-F^* mice relative to age-matched littermate controls at seven months [6.5 ± 0.8 vs 3.0 ± 0.2 microglia per field of view (FOV; equal to 1.8 × 10^−2^ mm^2^)] and at 24 months (15.9 ± 3.8 vs 2.8 ± 0.8 to); significant differences were found between time points and genotypes (Fig. 1*A*,*D*,*G*; two-way ANOVA, *p* = 0.0101 and *p* = 0.0002, respectively^a^). Iba1 area was also significantly elevated in transgenic mice compared with littermate controls, both at seven months (1505.8 ± 135.0 vs 821.9 ± 239.3 μm^2^) and 24 months [1982.4 ± 471.6 vs 781.7 ± 121.1 μm^2^; Fig. 1*A*,*E*; two-way ANOVA, *p* = 0.1821 (time points) and *p* = 0.0018 (genotypes)^b^]. To assess microglial *Dkk2* expression levels, we quantified *Dkk2* mRNA FISH signal that was colocalized with Iba1 immunoreactivity (Fig. 1*C*). The normalized area of *Dkk2* signal per DAPI^+^/Iba1^+^ microglial cell reached significantly higher levels in *APP^NL-G-F^* mice relative to littermate controls, both at seven months (0.3 ± 0.2 vs 0.1 ± 0.1 μm^2^) and at 24 months [1.2 ± 0.4 vs 0.1 ± 0.1 μm^2^; Fig. 1*A*,*F*,*G*; two-way ANOVA, *p* = 0.0245 (time points) and *p* = 0.0013 (genotypes)^c^].

10.1523/ENEURO.0306-22.2022.tab6-1 Extended Data Table 6-1 Human sample demographic data. Related to Figure 6. Table listing demographic data of individual subjects contributing to the generation of dataset in Figure 6. Clinical presentation as well as postmortem brain assessments are shown [brain weight, postmortem (PM) delay, Braak & Braak stage, CERAD score, THAL stage, and ABC score]. Download Table 6-1, DOCX file.

10.1523/ENEURO.0306-22.2022.tab6-2 Extended Data Table 6-2 mRNA FISH signal detection parameters. Related to Materials and Methods as well as Figure 6. Signal detection parameters used to identify *DKK2*, *TREM2*, and *P2RY12* mRNA FISH signal on confocal images form human samples using HALO software with the FISH-IF v2.0.4 module (Indica Labs). Download Table 6-2, DOCX file.

10.1523/ENEURO.0306-22.2022.f1-1 Extended Data Figure 1-1 Microglial *Dkk2* upregulation in *APP^NL-G-F^* mice – % *Dkk2^+^* microglia. Related to Figure 1. Relative contribution (%) of *Dkk2^+^* microglia versus the total microglia population in the motor cortex (***A***) and CA1 hippocampus (***B***) of *APP^NL-G-F^* mice as assessed by *Dkk2* mRNA FISH as well as microglial Iba1 IHC labelling. Individual data points represent the average of four FOVs analyzed for each animal. *N* = 4 animals per condition and time point, *n* = 4 different fields of view/animal and brain region. Two-way ANOVA with multiple comparisons test. *p < 0.05, **p < 0.01, ***p < 0.001, ****p < 0.0001 (^g, h^). Download Figure 1-1, TIF file.

Similar patterns of microgliosis and *Dkk2* upregulation were observed in the hippocampal CA1 region. Microglia count numbers were markedly elevated in *APP^NL-G-F^* mice compared with littermate controls both at seven months [3.0 ± 1.1 vs 2.6 ± 0.4 (n.s.)] and at 24 months [10.4 ± 2.3 vs 2.7 ± 0.5; Fig. 1*B*,*H*,*K*; mixed-effects analysis, *p* = 0.0005 (time points) and *p* = 0.0051 (genotypes)^d^]. Accordingly, detected Iba1 area was also increased: 1072.6 ± 146.3 versus 846.9 ± 292.8 μm^2^ at seven months and 1997.7 ± 511.8 versus 970.6 ± 221.3 μm^2^ at 24 months [Fig. 1*B*,*J*; two-way ANOVA, *p* = 0.0119 (time points) and *p* = 0.0288 (genotypes)^e^]. *Dkk2* expression per microglial cell quantified by mRNA FISH remained unchanged between *APP^NL-G-F^* mice and littermate controls at seven months (0.2 ± 0.1 vs 0.2 ± 0.1 μm^2^) but was higher at 24 months [0.6 ± 0.4 vs 0.1 ± 0.0 μm^2^; Fig. 1*B*,*J*,*K*; two-way ANOVA, *p* = 0.1363 (time points) and *p* = 0.0652 (genotypes)^f^].

Finally, we also assessed whether there were genotype-related changes in the relative contribution of *Dkk2^+^* microglia versus the total microglia population. In the cortex, the percentage of *Dkk2^+^* microglia was significantly elevated in *APP^NL-G-F^* mice compared with littermate controls both at seven months (48.1 ± 22.3 vs 18.8 ± 5.1%) and at 24 months [84.1 ± 5.1 vs 15.6 ± 7.7%; Extended Data Fig. 1-1*A*; two-way ANOVA, *p* = 0.0240 (time points) and *p* = 0.0004 (genotypes)^g^]. In the CA1 hippocampus, the percentage of *Dkk2^+^* microglia was similarly elevated in *APP^NL-G-F^* mice compared with littermate controls at seven months [34.9 ± 16.9 vs 7.8 ± 9.4%) and at 24 months (58.4 ± 21.6 vs 18.4 ± 17.7%; Extended Data Fig. 1-1*B*; two-way ANOVA, *p* = 0.0784 (time points) and *p* = 0.0093 (genotypes)^h^].

Taken together, our data demonstrate robust microgliosis in conjunction with *Dkk2* upregulation in *APP^NL-G-F^* mice compared with littermate controls, adding a spatial dimension to a previously published single-cell RNA sequencing (RNA-Seq) study that identified *Dkk2* expression in DAM/ARM microglia of the same mouse model (Sala Frigerio et al., 2019).

### Microgliosis and microglial *Dkk2* upregulation in *APP/PS1* mice

Following investigation of *APP^NL-G-F^* mice, we assessed microgliosis and *Dkk2* upregulation in a second AD mouse model, the *APP/PS1* mouse, that expresses chimeric mutant mouse/human *App* and mutant human *presenilin 1*, both associated with early onset familial AD in humans (Jankowsky et al., 2004).

**Figure 2.**
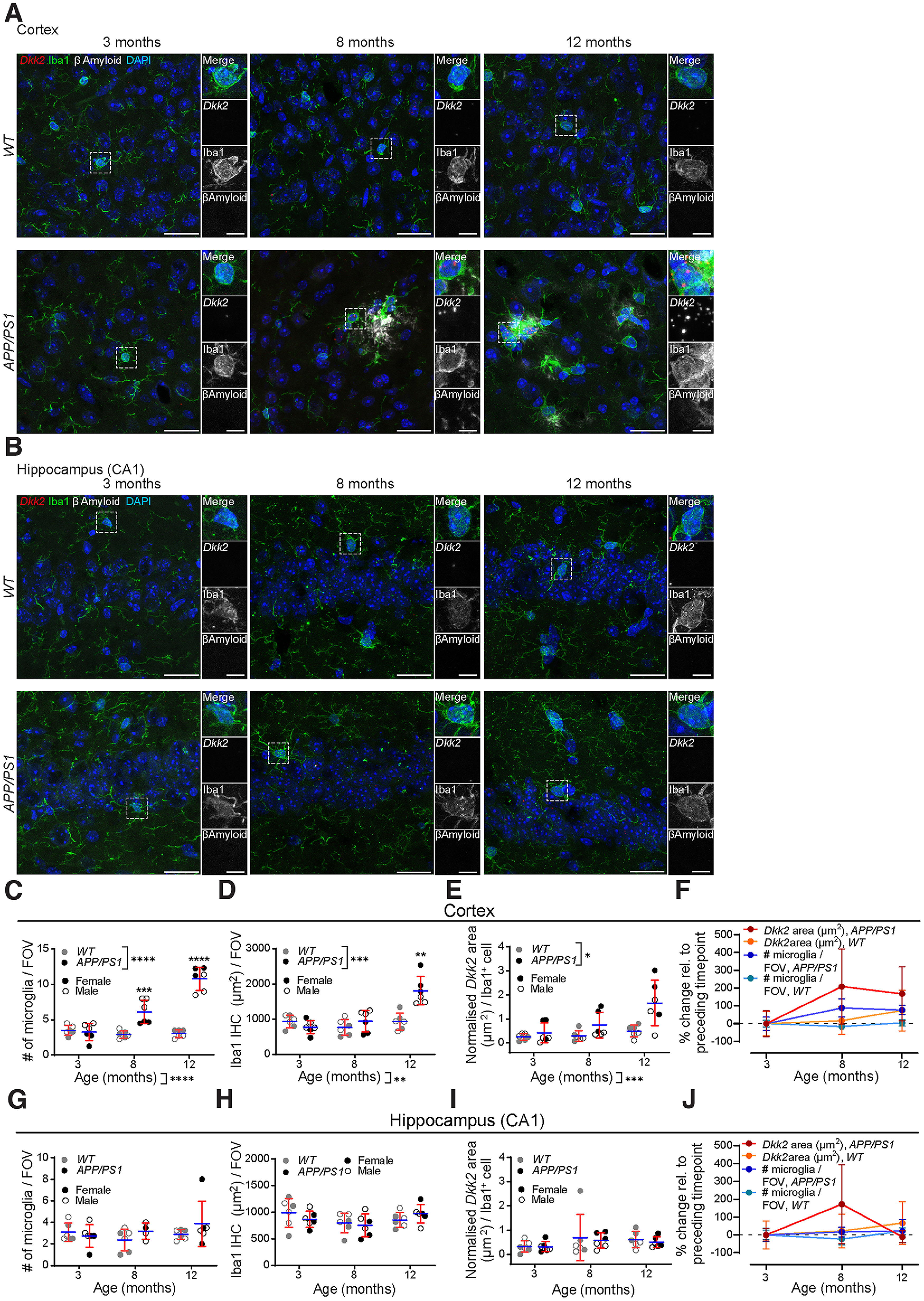
Microgliosis and microglial *Dkk2* upregulation in *APP/PS1* vs. wild-type (*WT*) mice. *Dkk2* mRNA. FISH as well as microglial Iba1 and βAmyloid IHC labeling in the motor cortex (***A***) and CA1 hippocampus (***B***) of *APP/PS1* mice. Boxed regions of interest (ROIs) were magnified for increased detail. ***C–F***, Microgliosis and *Dkk2* expression quantification in the *APP/PS1* motor cortex. ***C***, Quantification of microglia numbers per maximum projected FOV (FOV = 1.8 × 10^2^ mm^2^). ***D***, Iba1 IHC surface area per maximum projected FOV. ***E***, Normalized *Dkk2* mRNA FISH signal area per DAPI^+^/Iba^+^ microglial cell. ***F***, Comparative % changes of *Dkk2* expression and microglia numbers during time course. ***G–J***, Microgliosis and Dkk2 expression quantification in the *APP/PS1* CA1 hippocampus. ***G***, Quantification of microglia numbers per maximum projected FOV. ***H***, Iba1 IHC surface area per maximum projected FOV. ***I***, Normalized *Dkk2* mRNA FISH signal area per DAPI^+^/Iba^+^ microglial cell. ***J***, Comparative % changes of *Dkk2* expression and microglia numbers during time course. Individual data points represent the average of four FOVs analyzed for each animal (***C–E***, ***G–I***) or total averages from all animals per group (***F***, ***J***). *N* = 6 animals (3× females, 3× males) per time point and condition, *n* = 4 different fields of view/animal and brain region. Scale bars: 25 μm (***A–C***) and 5 μm (magnified ROIs). Data show mean +/– SD. Two-way ANOVA with multiple comparisons test. *p < 0.05, **p < 0.01, ***p < 0.001, ****p < 0.0001 (^i–k^). See also Extended Data Figure 2-1.

βAmyloid plaque load progressively increased in *APP/PS1* mice starting from eight months, whereas age-matched wild-type control littermates lacked βAmyloid plaques altogether. This was especially evident in the cortex (Fig. 2*A*). While plaques were detectable in the hippocampus of *APP/PS1* mice (data not shown), CA1 stratum pyramidale proximal regions, the standardized hippocampal brain region that was imaged in our study, rarely exhibited plaque depositions (Fig. 2*B*).

10.1523/ENEURO.0306-22.2022.f2-1 Extended Data Figure 2-1 Microglial *Dkk2* upregulation in *APP/PS1* mice – % *Dkk2^+^* microglia. Related to Figure 2. Relative contribution (%) of *Dkk2^+^* microglia versus the total microglia population in the motor cortex (***A***) and CA1 hippocampus (***B***) of *APP/PS1* mice as assessed by *Dkk2* mRNA FISH as well as microglial Iba1 IHC labelling. Individual data points represent the average of four FOVs analyzed for each animal. *N* = 6 animals (3× females, 3× males) per time point and condition, *n* = 4 different fields of view/animal and brain region. Two-way ANOVA with multiple comparisons test. *p < 0.05, **p < 0.01, ***p < 0.001, ****p < 0.0001 (^l, m^). Download Figure 2-1, TIF file.

While the number of DAPI^+^/Iba1^+^ microglia remained unchanged in *APP/PS1* mice versus littermate controls at three months (3.5 ± 0.6 to 3.3 ± 1.1 per FOV), their counts were significantly higher in *APP/PS1* relative to control mice at eight months (6.1 ± 1.5 vs 2.9 ± 0.5) and at 12 months [10.8 ± 1.5 vs 3.0 ± 0.5 to; Fig. 2*A*,*C*; two-way ANOVA, *p* < 0.0001 (time points) and *p* < 0.0001 (genotypes)^i^]. Iba1 area did not markedly differ between transgenic and littermate control mice at three months (774.8 ± 175.2 vs 935.4 ± 164.0 μm^2^) and at eight months (945.1 ± 275.8 vs 764.1 ± 205.3 μm^2^) but was significantly elevated in *APP/PS1* mice at 12 months [1811.1 ± 367.9 vs 937.9 ± 220.8 μm^2^; Fig. 2*A*,*D*; two-way ANOVA, *p* = 0.0030 (time points) and *p* = 0.0006 (genotypes)^j^]. Microgliosis in *APP/PS1* mice was accompanied by progressively increasing *Dkk2* expression per microglial cell at the mRNA level (1.2 ± 0.9, 3.6 ± 2.5, and 9.7 ± 5.5 μm^2^ at 3/8/12 months), whereas this metric remained unchanged in age-matched littermate controls [0.9 ± 0.6, 1.0 ± 0.7, and 1.8 ± 1.2 μm^2^; Fig. 2*A*,*E*; two-way ANOVA, *p* = 0.0003 (time points) and *p* = 0.0391 (genotypes)^k^]. The rate of increase of microgliosis (number of microglia) and *Dkk2* expression in *APP/PS1* mice was rapid between the ages of three and eight months (88.3 ± 46.9% for microgliosis, 208.6 ± 192.9% for *Dkk2* expression), at which point, it plateaued (76.3 ± 24.6% for microgliosis, 167.9 ± 138.2% for *Dkk2* expression; Fig. 2*A*,*F*). In agreement with published literature (Wang et al., 2003), we further noted that for the quantified metrics described above, female *APP/PS1* mice usually exhibited a more severe phenotype, especially at the final 12-month time point (Fig. 2*C–E*).

As noted above, hippocampal CA1 stratum pyramidale proximal regions in *APP/PS1* mice were mostly devoid of βAmyloid plaques. Here, we were unable to detect any changes in the number of DAPI^+^/Iba1^+^ microglia (Fig. 2*B*,*G*,*J*), Iba1 area (Fig. 2*B*,*H*), and *Dkk2* mRNA signal per microglial cell compared with age-matched littermate controls (Fig. 2*B*,*I*,*J*; two-way ANOVA, all n.s.).

Finally, unlike in *APP^NL-G-F^* mice, we could not detect any significant time point-related changes in the relative contribution of *Dkk2^+^* microglia versus the total microglia population in *APP/PS1* mice compared with littermate controls both in the cortex [Extended Data Fig. 2-1*A*; two-way ANOVA, *p* = 0.3563 (time points) and *p* = 0.7931 (genotypes)^l^] and in the CA1 hippocampus [Extended Data Fig. 2-1*B*; two-way ANOVA, *p* = 0.1691 (time points) and *p* = 0.7041 (genotypes)^m^].

Thus, we were able to largely replicate our findings regarding microgliosis and microglial *Dkk2* upregulation in two widely used AD mouse models (*APP^NL-G-F^* and *APP/PS1* mice), again adding spatial information to a previously published meta-analysis of single-cell RNA-Seq datasets (Friedman et al., 2018). However, the lack of βAmyloid plaques and microglial phenotype in hippocampal CA1 stratum pyramidale proximal regions of the *APP/PS1* mouse evokes the notion that the microglial phenotype investigated here could be linked to plaque proximity.

### *Dkk2^+^* microglia exhibit increased propensity for clustering around βAmyloid plaques

**Figure 3.**
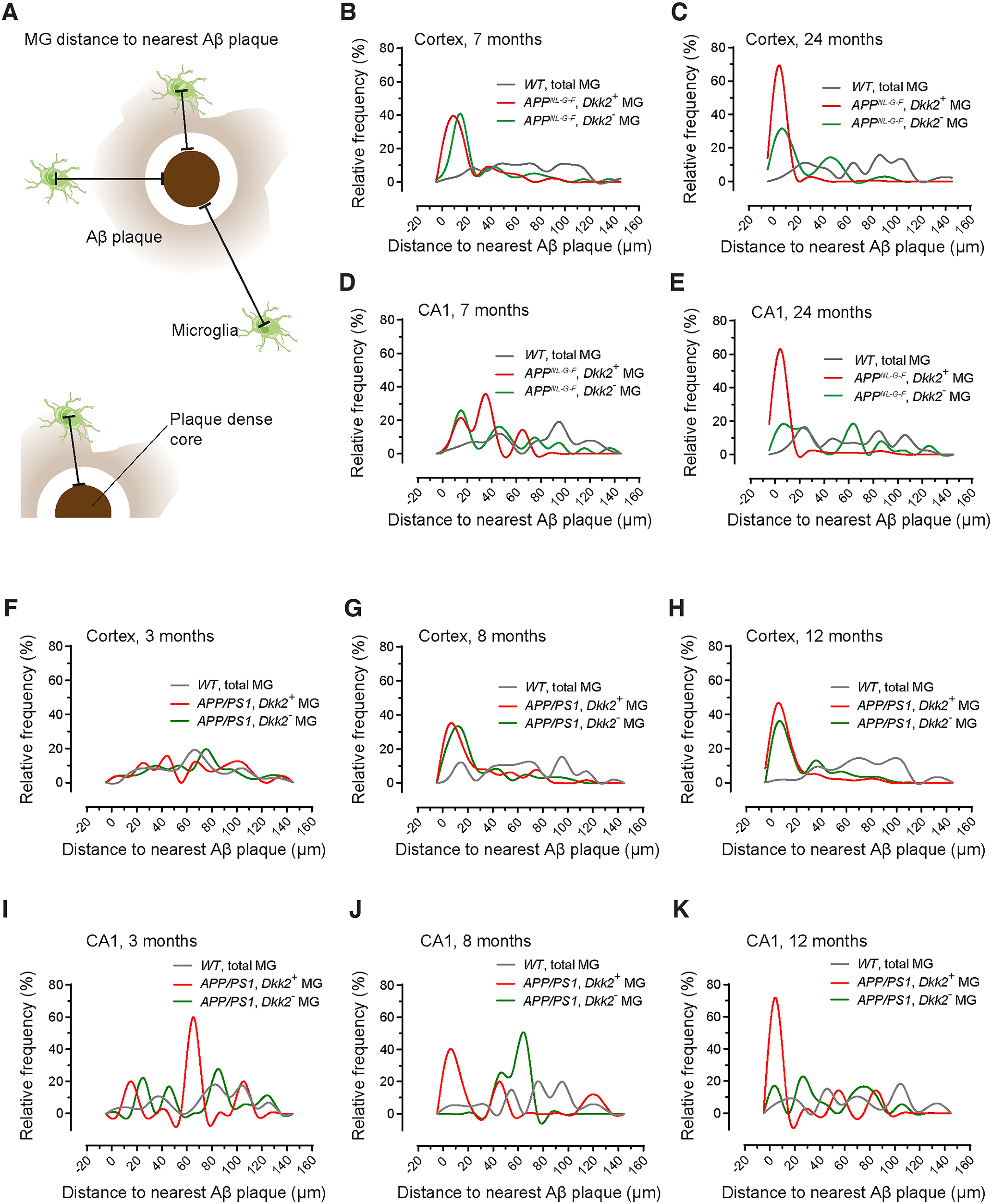
*Dkk2^+^* microglia cluster around βAmyloid plaques in *APP^NL-G-F^* and *APP/PS1* vs. wild-type (*WT*) mice. ***A***, Schematic showing methodology of measuring distances between microglia and nearest βAmyloid plaque dense core. ***B–E***, Distribution of microglia [*Dkk2^+^*, *Dkk2^–^*, or total microglia (MG) populations] distances to nearest βAmyloid plaque dense core in *APP^NL-G-^*^F^ or littermate control mice. Relative frequency distribution in the *APP^NL-G-^*^F^ or control motor cortex at seven months (***B***) and 24 months (***C***). Relative frequency distribution in the *APP^NL-G-^*^F^ or control CA1 hippocampus at seven months (***B***) and 24 months (***C***). ***F–K***, Distribution of microglia [*Dkk2^+^*, *Dkk2^–^*, or total microglia (MG) populations] distances to nearest βAmyloid plaque dense core in *APP/PS1* or littermate control mice. Relative frequency distribution in the *APP/PS1* or control motor cortex at three months (***F***), eight months (***G***), and 12 months (***H***). Relative frequency distribution in the *APP/PS1* or control CA1 hippocampus at three months (***I***), eight months (***J***), and 12 months (***K***). *APP^NL-G-^*^F^/control: *N* = 4 animals per condition and time point, *n* = 4 different fields of view/animal and brain region; *APP/PS1*/control: *N* = 6 animals (3× females, 3× males) per time point and condition, *n* = 4 different fields of view/animal and brain region. See also Extended Data Figure 3-1.

To investigate whether *Dkk2* expression status was correlated with βAmyloid plaque proximity, we performed nearest neighbor analysis to quantify the spatial relationship between microglia and the nearest βAmyloid plaque dense core identified following βAmyloid IHC in *APP^NL-G-F^* and *APP/PS1* mice (schematic shown in Fig. 3*A*). Frequency distributions of recorded distances were summarized in histograms. In AD mouse models, we distinguished between *Dkk2^+^* and *Dkk2^–^* microglia, whereas no such distinction was made in wild-type mice as *Dkk2* expression levels were negligible at all time points (Figs. 1*A*,*B*,*F*,*J*, 2*A*,*B*,*E*,*I*). Furthermore, where no plaques were evident (e.g., in wild-type or predisease stage mice or in some hippocampal CA1 stratum pyramidale proximal regions) distances to plaque dense core “placeholders” randomly placed on confocal images were measured instead.

10.1523/ENEURO.0306-22.2022.f3-1 Extended Data Figure 3-1 Statistical analysis for microglial clustering around βAmyloid plaques. Related to Figure 3. ***A–H***, Skewness and kurtosis analysis of histograms from microglia-βAmyloid plaque nearest neighbor analysis on *APP^NL-G-F^* mice in Figure 3 in the cortex (***A–D*** and CA1 hippocampus (***E–H***) as well as at seven months (***A***, ***B***, ***E***, ***F***) and at 24 months (***C***, ***D***, ***G***, ***H***). ***I–T***, Skewness and kurtosis analysis of histograms from microglia-βAmyloid plaque nearest neighbor analysis on *APP/PS1* mice in Figure 3 in the cortex (***I–N***) and CA1 hippocampus (***O–T***) as well as at three months (***I***, ***J***, ***O***, ***P***), eight months (***K***, ***L***, ***Q***, ***R***), and at 12 months (***M***, ***N***, ***S***, ***T***). Data points represent mean values for individual analyzed animals. *APP^NL-G-^*^F^/control: *N* = 4 animals per condition and time point, *n* = 4 different fields of view/animal and brain region; *APP/PS1*/control: *N* = 6 animals (3× females, 3× males) per time point and condition, *n* = 4 different fields of view/animal and brain region. One-way ANOVA with Tukey’s *post hoc* test. *p < 0.05, **p < 0.01, ***p < 0.001, ****p < 0.0001 (^n–p^). Download Figure 3-1, TIF file.

As would be expected, wild-type littermate controls of *APP^NL-G-F^* mice used in our study exhibited microglia at varying/random distances to the nearest randomly assigned dense core placeholder in the motor cortex and CA1 hippocampus at 7 and 24 months (Figs. 1*A*,*B*, 3*B–E*). This finding is in keeping with the homogeneous tiling behavior usually exhibited by microglia in the healthy CNS (Nimmerjahn et al., 2005). In stark contrast, a large proportion of *Dkk2^+^* and *Dkk2^–^* microglia were found within 20 μm of the nearest plaque dense core in the cortex of seven-month-old *APP^NL-G-F^* mice, while *Dkk2^+^* microglia were predominantly located within 40 μm of plaque dense cores in the CA1 hippocampus (Fig. 3*B*,*D*). By 24 months, the clustering of microglia around βAmyloid plaque dense cores, especially that of *Dkk2^+^* microglia, became even more pronounced both in the cortex and in the CA1 hippocampus (Fig. 3*C*,*D*). Skewness and kurtosis analyses of histogram distribution curves for each individual animal revealed that *APP^NL-G-F^* microglia were statistically significantly more tightly clustered around plaque dense cores with increasing age than microglia of age-matched control mice (Extended Data Fig. 3-1*A–H*). Crucially, however, in 24-month-old *APP^NL-G-F^* mice, *Dkk2^+^* microglia were statistically significantly more tightly associated with plaques than *Dkk2^–^* microglia both in the cortex and in the CA1 hippocampus (Extended Data Fig. 3-1*C*,*D*,*G*,*H*; one-way ANOVA^n^).

We observed similar plaque-microglia distance relationships in *APP/PS1* mice and respective wild-type littermate controls. Microglia in the wild-type littermate control mouse cortex and CA1 hippocampus were evenly distributed relative to the nearest randomly placed plaque dense core placeholder (Fig. 3*F–K*). In the cortex of three-month-old (predisease stage and plaque free) *APP/PS1* mice, both *Dkk2^+^* and *Dkk2^–^* microglia exhibited similar wild-type-like distance distributions (Fig. 3*F*), whereas microglia increasingly clustered within 20 μm of plaque dense cores at subsequent (disease stage) time points, with *Dkk2^+^* microglia exhibiting slightly more pronounced clustering versus *Dkk2^–^* microglia at 12 months (Fig. 3*G*,*H*); we note that the latter difference was not statistically significant according to skewness and kurtosis analysis, while clustering of *Dkk2^+^* microglia around plaques dense cores in *APP/PS1* mice was statistically significantly increased versus that of microglia of age-matched control mice from eight months onwards (Extended Data Fig. 3-1*I–N*; one-way ANOVA^o^). As discussed above, because of the small amounts of βAmyloid plaques in hippocampal CA1 stratum pyramidale proximal regions of the *APP/PS1* mouse, microglia distributions were comparatively variable, especially at three months (Fig. 3*I*), although substantial clustering of *Dkk2^+^* microglia was registered in those instances were βAmyloid plaques were observed in CA1 stratum pyramidale proximal regions at 8 and 12 months (Fig. 3*J*,*K*). Accordingly, skewness and kurtosis analyses were inconclusive for CA1 microglia (Extended Data Fig. 3-1*O–T*; one-way ANOVA^p^).

While it is widely known that microglia accumulate around CNS lesions such as βAmyloid plaques, our data further suggest that clustering around plaques is frequently accompanied by the expression of *Dkk2*, especially in the *APP^NL-G-F^* AD mouse model. Conversely, in the healthy brain, microglia were evenly tiled and lacked *Dkk2* expression.

### Microgliosis and microglial *Dkk2* upregulation in *SOD1^G93A^* ALS mice

Having demonstrated microgliosis and clustering of *Dkk2^+^* microglia around βAmyloid plaques in two different widely used AD mouse models, we next investigated whether our findings could be recapitulated in another neurodegeneration mouse model, the *SOD1^G93A^* amyotrophic lateral sclerosis (ALS) mouse (Gurney et al., 1994). According to the meta-analysis of single-cell RNA-Seq datasets by Friedman et al., 2018; microglial *Dkk2* upregulation should also be evident in this mouse model. It expresses the mutant human *SOD1^G93A^* gene that causes motor neuron degeneration in the spinal cord and other parts of the CNS, which underlies ALS (Gurney et al., 1994). We performed mRNA FISH to detect microglial *Dkk2* mRNA *in situ* paired with immunohistochemical labeling using an antibody against Iba1 on transverse cryosections from the lumbar (L)5 region of the spinal cord and acquired images from the ventral horn, an area that displays robust motor neuron degeneration in this mouse model (Gurney et al., 1994).

**Figure 4.**
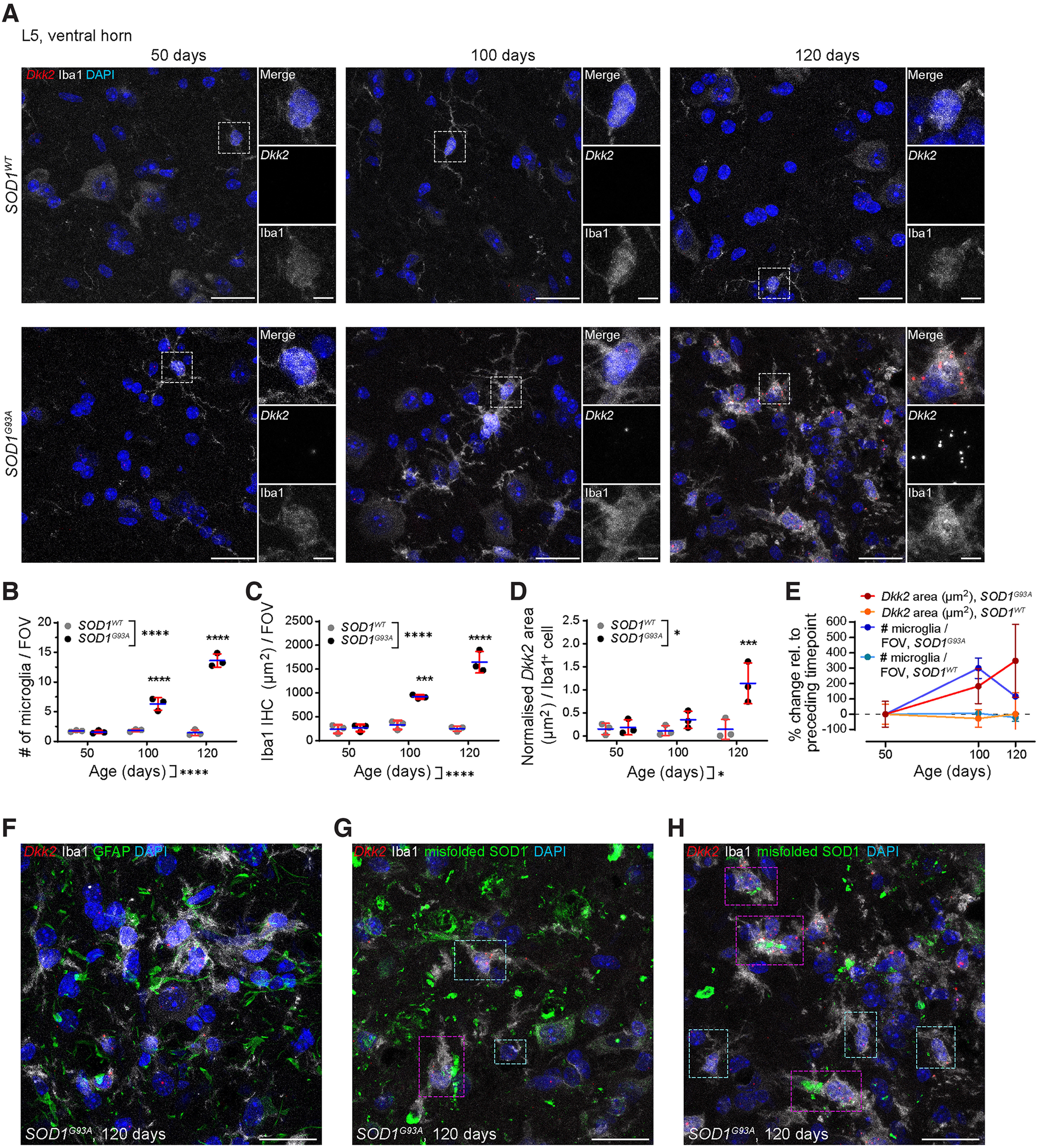
Microgliosis and microglial *Dkk2* upregulation in *SOD1^G93A^* AL*S* vs. wild-type (*WT*) mice. ***A***, *Dkk2* mRNA FISH and microglial Iba1 IHC labeling in the L5 spinal cord ventral horn of mice transgenically expressing human *SOD1^WT^* (control) or mutant *SOD1^G93A^* at 50, 100, and 120 d. Boxed regions of interest (ROIs) were magnified for increased detail. ***B***, Quantification of microglia numbers per maximum projected FOV (FOV = 1.8 × 10^−2^ mm^2^). ***C***, Iba1 IHC surface area per maximum projected FOV. ***D***, Normalized *Dkk2* mRNA FISH signal area per DAPI^+^/Iba^+^ microglial cell. ***E***, Comparative % changes of *Dkk2* expression and microglia numbers during time course. ***F***, *Dkk2* mRNA FISH together with microglial Iba1 and astroglial GFAP IHC labeling in the L5 spinal cord ventral horn of 120-d-old *SOD1^G93A^* mice. ***G***, ***H***, *Dkk2* mRNA FISH together with microglial Iba1 and misfolded SOD1 IHC labeling in the L5 spinal cord ventral horn of 120-d-old *SOD1^G93A^* mice. Magenta and cyan ROIs, respectively, depict proximity and absence of clear association between DAPI^+^/Iba1^+^ microglia and misfolded SOD1 foci. Individual data points represent the average of four FOVs analyzed for each animal (***C***, ***D***) or total averages from all animals per group (***E***). *N* = 3 animals per time point and condition, *n* = 4 fields of view per animal. Scale bars: 25 μm (***A***, ***F–H***) and 5 μm (magnified ROIs). Data show mean +/– SD. Two-way ANOVA with multiple comparisons test. *p < 0.05, **p < 0.01, ***p < 0.001, ****p < 0.0001 (^q–s^). See also Extended Data Figure 4-1.

In control mice expressing wild-type human *SOD1 (SOD1^WT^)*, but not in age-matched mice expressing *SOD1^G93A^*, no overt changes in microgliosis and *Dkk2* expression were observed at any of the assessed time points (Fig. 1*A–E*). At the early 50-d time point, *SOD1^G93A^* mice still exhibited control levels of microgliosis [1.7 ± 0.1 vs 1.6 ± 0.2 DAPI^+^/Iba1^+^ microglia per FOV (Fig. 4*A*,*B*), 244.7 ± 73.2 vs 266.5 ± 59.7 μm^2^ Iba1 area (Fig. 4*A*,*C*)]. However, the number of microglia was significantly elevated in *SOD1^G93A^* compared with age-matched control *SOD1^WT^* mice at 100 d (6.3 ± 0.9 vs 1.8 ± 0.2) and at 120 d [13.6 ± 0.9 vs 1.5 ± 0.4; Fig. 4*A*,*B*; two-way ANOVA, *p* < 0.0001 (time points) and *p* = 0.0001 (genotypes)^q^]. Accordingly, the area of Iba1 immunoreactivity was also significantly higher in *SOD1^G93A^* versus *SOD1^WT^* mice at 100 d (918.8 ± 31.7 vs 328.1 ± 75.3 μm^2^) and at 120 d [1646.0 ± 184.7 versus 248.7 ± 42.1 μm^2^; Fig. 4*A*,*C*; two-way ANOVA, *p* < 0.0001 (time points) and *p* = 0.0001 (genotypes)^r^]. *Dkk2* expression per microglial cell progressively increased in *SOD1^G93A^* but not in *SOD1^WT^* mice, although this increase only reached significance at 120 d: 0.8 ± 0.7, 2.4 ± 0.8, and 10.7 ± 4.6 μm^2^ in *SOD1^G93A^* mice at 50/100/120 d; 0.7 ± 0.5, 0.5 ± 0.3, and 0.5 ± 0.6 μm^2^ in *SOD1^WT^* mice at 50/100/120 d [Fig. 4*A*,*D*; two-way ANOVA, *p* = 0.0154 (time points) and *p* = 0.0193 (genotypes)^s^]. Thus, fast-paced microgliosis is evident between 50 and 100 d in *SOD1^G93A^* mice, with a slightly reduced rate of acceleration between 100 and 120 d (Fig. 4*E*). Conversely, microglial *Dkk2* upregulation appears to accelerate especially in the final pathologic stages. This resulted in a relative contribution of *Dkk2^+^* microglia versus total microglia that was significantly increased in *SOD1^G93A^* compared with *SOD1^WT^* mice: 45.4 ± 5.1, 56.5 ± 19.2, and 75.0 ± 4.4% in *SOD1^G93A^* mice at 50/100/120 d; 39.9 ± 17.4, 27.5 ± 14.3, and 14.6 ± 3.6% in *SOD1^WT^* mice at 50/100/120 d [Extended Data Fig. 4-1*A*; two-way ANOVA, *p* = 0.9125 (time points) and *p* = 0.0073 (genotypes)^t^].

10.1523/ENEURO.0306-22.2022.f4-1 Extended Data Figure 4-1 Microglial *Dkk2* upregulation in *SOD1^G93A^* AL*S* mice – % *Dkk2^+^* microglia. Related to Figure 4. Relative contribution (%) of *Dkk2^+^* microglia versus the total microglia population in the L5 ventral horn spinal cord of *SOD1^G93A^* AL*S* mice as assessed by *Dkk2* mRNA FISH as well as microglial Iba1 IHC labelling. Individual data points represent the average of four FOVs analyzed for each animal. *N* = 3 animals per time point and condition, *n* = 4 fields of view per animal. Two-way ANOVA with multiple comparisons test. *p < 0.05, **p < 0.01, ***p < 0.001, ****p < 0.0001 (^t^). Download Figure 4-1, TIF file.

We next sought to investigate whether microgliosis and microglial *Dkk2* upregulation in the *SOD1^G93A^* ALS mouse model were spatially correlated with local CNS lesions, analogous to that observed in the *APP^NL-G-F^* and *APP/PS1* AD mouse models. In absence of AD-typical βAmyloid plaques in ALS, we combined *Dkk2* mRNA FISH and microglial immunohistochemical labeling with the immunolabelling of GFAP to visualize astrocytes and immunolabelling of misfolded SOD1 to visualize aggregates of misfolded mutant SOD1^G93A^. In 120-d-old *SOD1^G93A^* ALS mice, we failed to detect clustering of microglia, regardless of their Dkk2 expression status, specifically around GFAP (Fig. 4*F*). However, we observed some degree of microglial clustering around misfolded SOD1 immunoreactivity [Fig. 4*G*,*H*; magenta regions of interest (ROIs)]. However, many microglia did not exhibit local accumulation around misfolded SOD1 lesions (Fig. 4G,*H*, cyan ROIs). In absence of a clear clustering pattern, these observations were not quantified.

Taken together, the microgliosis and microglial *Dkk2* upregulation detected in the brains of AD mouse models could also be replicated in an unrelated neurodegeneration mouse model, namely in the spinal cord of the *SOD1^G93A^* ALS mice. While some degree of clustering around misfolded SOD1 aggregates occurred, this was not as robust as the clustering around βAmyloid plaques in the *APP^NL-G-F^* and *APP/PS1* AD mouse models. Nonetheless, our findings support the published notion that *Dkk2* upregulation may be part of a general response in CNS microglia as they transition from surveillance to activation (DAM/ARM microglia), at least in mouse models of neurodegeneration (Friedman et al., 2018; Sala Frigerio et al., 2019; Meilandt et al., 2020). This supports the possibility that *Dkk2* represents a DAM/ARM marker gene, at least in mice.

### DKK2 recombinant protein disrupts WNT7a-induced synapse features in cultured neurons

We next sought to investigate what effect Dkk2 protein secreted by microglia might have on its surroundings under the assumption that increased microglial *Dkk2* expression at the mRNA level results in increased microglial Dkk2 protein secretion. We focused our study on synapses in mature primary neuron cultures because of the well-known anti-synaptic effect that the Dkk2 homolog Dkk1 has on them, which it brings about by decreasing canonical and increasing noncanonical Wnt signaling (Purro et al., 2012; Galli et al., 2014; Marzo et al., 2016; Elliott et al., 2018; Sellers et al., 2018). However, we note that, in principle, Dkk2 can have context-dependent agonistic and antagonistic effects (Mao and Niehrs, 2003).

**Figure 5.**
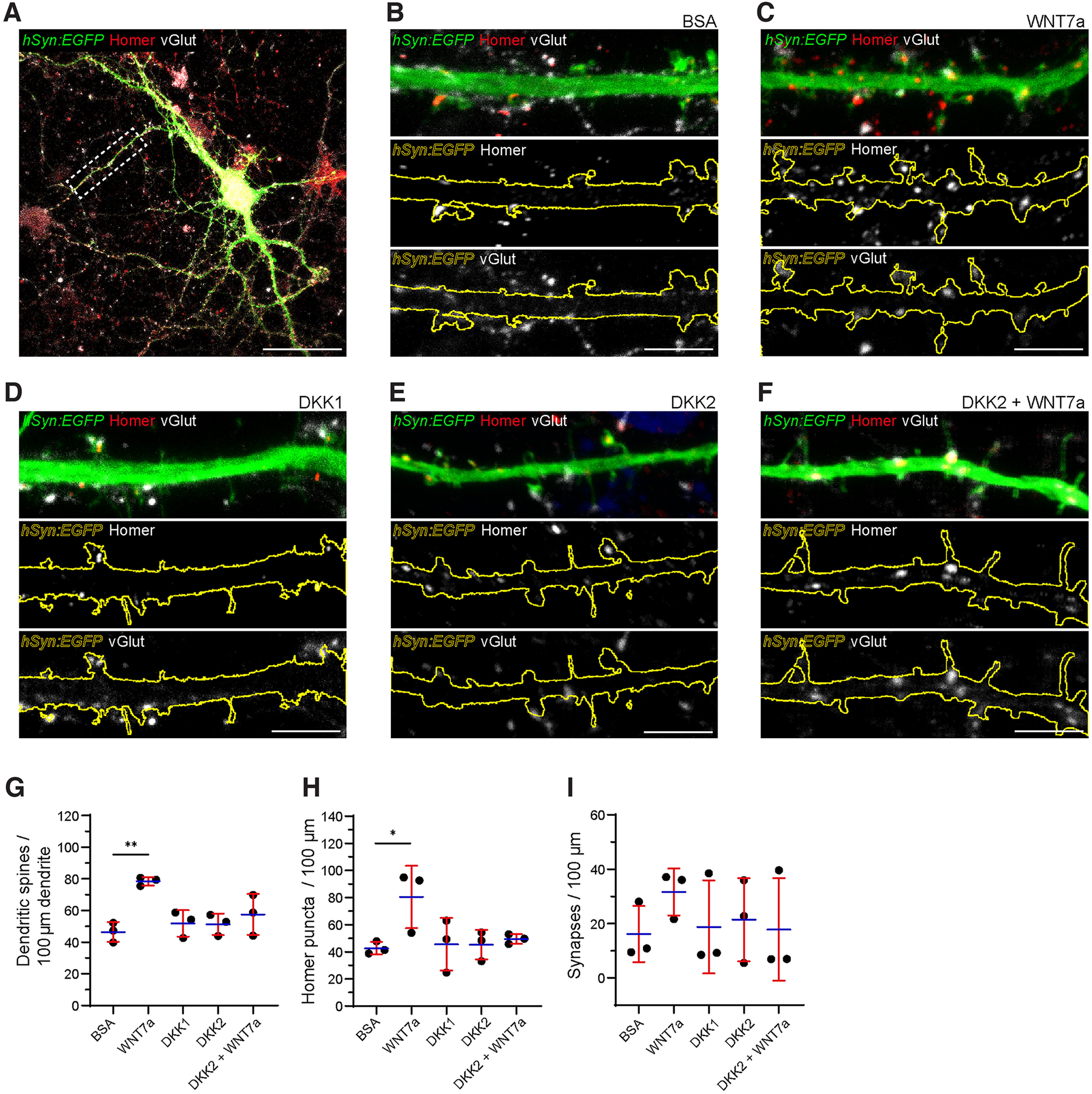
Recombinant DKK2 protein neutralises the synaptogenic effect of WNT7a in mature hippocampal primary neurons. ***A***, Typical rat hippocampal neuron at DIV22 expressing *hSyn:EGFP* immunolabelled with Homer and vGlut. Boxed ROI indicates a primary dendritic branch, on which analysis in this section was focused. ***B–F***, Representative primary dendrites of DIV22 hippocampal neurons treated for 24 h with 100 ng/ml BSA control (***B***), 200 ng/ml WNT7a (***C***), 100 ng/ml DKK1 (***D***), 100 ng/ml DKK2 (***E***), and 100 ng/ml/200 ng/ml DKK2 + WNT7a (***F***). Immunolabelling for the presynaptic and postsynaptic markers vGlut and homer was performed, and merged views are shown in top panels. Remaining panels show homer (middle panel) and vGlut (lower panel) with outlined primary dendrite boundaries based on *hSyn:EGFP* labeling. ***G***, Normalized number of dendritic spines per 100-μm primary dendrite. ***H***, Normalized number of homer puncta per 100-μm primary dendrite. ***I***, Normalized number of synapses (defined as homer/vGlut apposition events with a maximum distance of 1 μm) per 100-μm primary dendrite. *N* = 3 biological repeats, *n* = at least 15 analyzed neurons per condition. Scale bars: 50 μm (***A***) and 5 μm (***B–F***). Data show mean +/– SD. One-way ANOVA with Tukey’s *post hoc* test. *p < 0.05, **p < 0.01 (^u, v^).

To this end, we treated mature rat hippocampal neuron cultures sparsely expressing *hSyn:EGFP* at 21 d *in vitro* with recombinant proteins for 24 h (WNT7a, 200 ng/ml; DKK1, 100 ng/ml; DKK2, 100 ng/ml; DKK2 + WNT7a, 100 and 200 ng/ml; BSA control, 100 ng/ml). Chosen recombinant protein concentrations were in line with published works and/or TCF/LEF dose dependence assays performed in house (data not shown). This was followed by immunocytochemical labeling using antibodies against the presynaptic and postsynaptic markers vGlut and Homer. A typical sparsely labeled (*hsyn:EGFP^+^*) neuron with highlighted primary dendrite (boxed ROI) that was used for analysis is depicted in Figure 5*A*. WNT7a treatment significantly increased the number of dendritic spines as well as the number of postsynaptic homer puncta compared with BSA treatment (Fig. 5*B*,*C*,*G*,*H*; one-way ANOVA, dendritic spines: *p* = 0.0023^u^; homer puncta: *p* = 0.0309^v^). Conversely, these metrics were unaffected by DKK1 and DKK2 treatment (Fig. 5*D*,*E*,*G*,*H*; one-way ANOVA; all n.s.) and crucially also by combined DKK2 + WNT7a treatment (Fig. 5*F–H*; one-way ANOVA; n.s.). The absolute number of synapses (defined as Homer/vGlut apposition events with up to 1 μm distance) was similarly increased by WNT7a but not by DKK1, DKK2 or a combination of DKK2 and WNT7a compared with BSA, although this did not reach statistical significance (Fig. 5*I*; one-way ANOVA; n.s.).

Nonetheless, these combined data suggest that DKK2 treatment is antagonistic rather than agonistic and completely abolishes the pro-synaptogenic effect of WNT7a treatment, at least in our *in vitro* assay. Furthermore, it appears that the antagonistic effect of DKK2 as well as that of the established Wnt signaling antagonist DKK1 rely on an inherent Wnt signaling tone that was low/absent in our cultures, as neither reduced synaptic metrics to levels below those found with BSA treatment when applied independently.

### *DKK2* is not upregulated in human microglia

We have thus far demonstrated significant microgliosis and microglial *Dkk2* upregulation in AD and ALS mouse models of neurodegeneration, as well as clustering of *Dkk2^+^* microglia around βAmyloid plaques. In combination with previously published studies, which have demonstrated microglial *Dkk2* upregulation by single-cell RNA-Seq (Friedman et al., 2018; Sala Frigerio et al., 2019; Meilandt et al., 2020), this led us to postulate that *Dkk2* may represent a bona fide DAM/ARM marker gene at least in neurodegeneration mouse models. We next sought to investigate whether our findings were recapitulated in human subjects diagnosed with AD.

**Figure 6.**
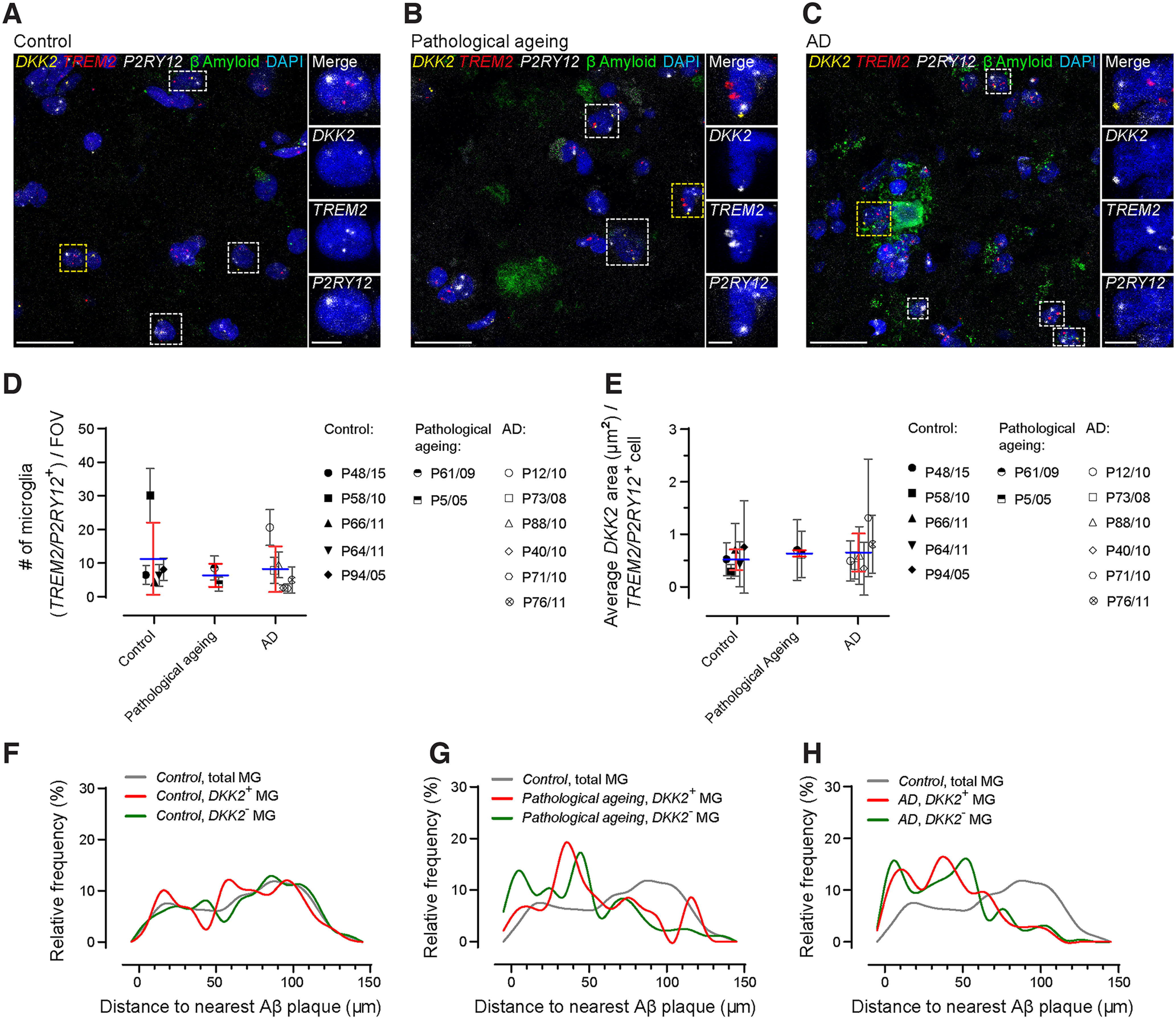
*DKK2* is not upregulated at the mRNA level in postmortem brains from AD patients. ***A–C***, Representative confocal images depicting microglial *DKK2* expression in the human frontal cortex. *DKK2* as well as microglial *TREM2* and *P2RY12* mRNA FISH signal in conjunction with βAmyloid IHC labeling in postmortem human frontal cortex samples from healthy control individuals (***A***), individuals diagnosed with pathologic aging (***B***), and individuals diagnosed with AD (***C***). Boxed ROIs highlight microglia expressing *DKK2* (DAPI^+^/*DKK2^+^*/*TREM2^+^*/*P2RY12^+^*); yellow boxed ROIs were enlarged for improved visualization. ***D***, Quantification of microglia (DAPI^+^/*TREM2^+^*/*P2RY12^+^*) numbers per maximum projected FOV (FOV = 1.8 × 10^−2^ mm^2^). ***E***, Normalized *DKK2* mRNA FISH signal area per DAPI^+^/*TREM2^+^*/*P2RY12^+^* microglial cell. ***F–H***, Distribution of DAPI^+^/*TREM2^+^*/*P2RY12^+^* microglia [*Dkk2^+^*, *Dkk2^–^*, or total microglia (MG) populations] distances to nearest βAmyloid plaque dense core in postmortem human frontal cortex samples. Individual plots show relative frequency distributions in individuals classified as healthy control (***F***), pathologic aging (***G***), and AD (***H***). Healthy control individuals: *N* = 5 individuals, *n* = 8 fields of view); AD (Braak & Braak stage 5–6): *N* = 6 individuals, *n* = 8 fields of view; pathologic aging (Braak & Braak stage 3–4): *N* = 2 individuals, *n* = 8 fields of view. Data points represent the average of four FOVs analyzed for each individual subject (mean ± SD); individual subject mean values were further averaged for each group of interest and summarized as mean ± SD (blue horizontal bars, red error bars). One-way ANOVA with Tukey’s *post hoc* test (^w, x^). No statistical differences identified. Scale bars: 25 μm (***A–C***) and 5 μm (***A–C*** enlarged ROIs). See also Extended Data Figure 6-1, Extended Data Tables 6-1 and 6-2.

To analyze microglial *DKK2* expression in humans, we obtained human postmortem frontal cortex brain tissue from healthy control individuals, as well as individuals diagnosed with AD and pathologic aging, the latter being defined as nondemented individuals with AD-typical histopathologic changes. Demographic data and postmortem brain assessments are summarized in Extended Data Table 6-1. We performed mRNA FISH to detect *DKK2* mRNA in microglia that were additionally labeled by mRNA FISH for the microglial markers *TREM2* and *P2RY12* (see also Extended Data Table 6-2 for added analysis parameters). This was paired with immunohistochemical labeling using an antibody against βAmyloid to label βAmyloid plaques. As expected, samples from control individuals were devoid of βAmyloid plaques while those classified “pathologic aging” and “AD” exhibited progressively increasing levels of plaque burden (Fig. 6*A–C*). However, we did not detect significant differences in the number of DAPI^+^/*TREM2^+^*/*P2RY12^+^* microglia per field of view between control, pathologic aging, and AD groups (Fig. 6*A–D*; control: 11.1 ± 10.7 microglia/FOV; pathologic aging: 6.2 ± 3.4; AD: 8.0 ± 6.8; one-way ANOVA, *p* = 0.4507^w^). This absence of microglia number changes is in line with findings from published literature (Marlatt et al., 2014; Davies et al., 2017; Paasila et al., 2019; Franco-Bocanegra et al., 2021). Similarly, *DKK2* expression per DAPI^+^/*TREM2^+^*/*P2RY12^+^* microglial cell did not differ between control (0.5 ± 0.2 μm^2^), pathologic aging (0.7 ± 0.1 μm^2^), and AD groups (0.7 ± 0.4 μm^2^; Fig. 6*A–C*,*E*; one-way ANOVA, *p* = 0.7689^x^). This was accompanied by unchanged relative contributions of *DKK2^+^* microglia across control (38.3 ± 9.7%), pathologic aging (38.3 ± 3.8%), and AD groups (41.6 ± 12.5%; Extended Data Fig. 6-1*A*; one-way ANOVA, *p* = 0.8650^y^). We further found that *DKK2* expression status had no effect on *TREM2* expression levels per DAPI^+^/*TREM2^+^*/*P2RY12^+^* microglial cell in control (*DKK2^+^*: 0.8 ± 0.3 μm^2^; *DKK2^–^*: 0.6 ± 0.3 μm^2^), pathologic aging (*DKK2^+^*: 0.9 ± 0.9 μm^2^; *DKK2^–^*: 0.7 ± 0.2 μm^2^), and AD individuals (*DKK2^+^*: 0.5 ± 0.2 μm^2^; *DKK2^–^*: 0.4 ± 0.2 μm^2^; Extended Data Fig. 6-1*B*; one-way ANOVA, *p* = 0.2349^z^). Conversely, *P2RY12* expression levels per DAPI^+^/*TREM2^+^*/*P2RY12^+^* microglial cell were increased in cells co-expressing *DKK2* compared with cells that lacked *DKK2* expression, although that difference was not statistically significant: control (*DKK2^+^*: 2.7 ± 1.0 μm^2^; *DKK2^–^*: 1.7 ± 0.5 μm^2^), pathologic aging (*DKK2^+^*: 2.7 ± 0.5 μm^2^; *DKK2^–^*: 2.0 ± 0.9 μm^2^), and AD individuals (*DKK2^+^*: 3.2 ± 1.6 μm^2^; *DKK2^–^*: 1.6 ± 0.8 μm^2^; Extended Data Fig. 6-1*C*; one-way ANOVA, *p* = 0.1056^ab^).

10.1523/ENEURO.0306-22.2022.f6-1 Extended Data Figure 6-1 *DKK2*, *TREM2*, and *P2RY12* expression the mRNA level in human postmortem brains. Related to Figure 6. ***A***, Relative contribution (%) of *DKK2^+^* microglia versus the total microglia population in the human postmortem frontal cortex of control, pathological ageing, and AD individuals as assessed by *DKK2*, *TREM2*, and *P2RY12* mRNA FISH. Normalized *TREM2* (***B***) and P2RY12 (***C***) mRNA FISH signal area per DAPI^+^/*TREM2^+^*/*P2RY12^+^* microglial cell in presence or absence of *DKK2* expression. Healthy control individuals: *N* = 5 individuals, *n* = 8 fields of view); AD (Braak & Braak stage 5–6): *N* = 6 individuals, *n* = 8 fields of view; pathological ageing (Braak & Braak stage 3–4): *N* = 2 individuals, *n* = 8 fields of view. Data points represent the average of 8 FOVs analyzed for each individual subject (mean ± SD for ***A***, mean for ***B***, ***C***); individual subject mean values were further averaged for each group of interest and summarized as mean ± SD (blue horizontal bars, red error bars). One-way ANOVA with Tukey’s *post hoc* test (^y, z, ab^). No statistical differences identified. Download Figure 6-1, TIF file.

We subsequently assessed the clustering behavior of microglia around βAmyloid plaques. In healthy control individuals, the total microglia population displayed a varying/random spatial distribution around the nearest randomly placed dense core placeholder, which furthermore did not appear to be modified by *DKK2* expression status (Fig. 6*A*,*F*). In individuals classified as “pathologic aging,” we identified emerging populations of both *DKK2^+^* and *DKK2^–^* microglia that frequently accumulated around βAmyloid plaque dense cores up to a distance of 50 μm, although clustering in the proximal most regions was more robust for *DKK2^–^* cells (Fig. 6*B*,*G*). This clustering was further consolidated, especially among *DKK2^+^* microglia, whose predominant distribution now also included proximal most regions (Fig. 6*C*,*H*).

Taken together, our data on human frontal cortex postmortem tissue indicate that neither the increase in microglial numbers nor microglial *DKK2* upregulation, both of which were evident in mouse models, occur in human brains under conditions classified as “pathologic aging” and “AD.” However, microglia did exhibit clustering behavior around βAmyloid plaques although this was not linked to *DKK2* expression.

## Discussion

Past and present research have linked dysregulated Wnt signaling to AD (Palomer et al., 2022; for previous review, see Purro et al., 2014; Palomer et al., 2019; Aghaizu et al., 2020; Inestrosa et al., 2021). However, recent research has also more intimately linked microglia and neuroinflammation to AD, as initially exemplified by variants of genes predominantly expressed in microglia like *TREM2* and *CD33* exhibiting disease modifying properties (Bradshaw et al., 2013; Guerreiro et al., 2013). New evidence even suggests that the microglial AD response is itself regulated by Wnt signaling, as the signaling pathway downstream of TREM2, essential for regulating microglial survival and proliferation, cross-talks with the Wnt pathway (Zheng et al., 2017; Meilandt et al., 2020).

Here, we sought to explore the role of *DKK2/Dkk2*, which encodes a Wnt signaling modulator, that was upregulated in a subpopulation of microglia (DAM/ARM) in various single and bulk cell RNA-Seq studies on neurodegeneration mouse models (Friedman et al., 2018; Sala Frigerio et al., 2019; Meilandt et al., 2020). Our histologic data obtained largely by mRNA FISH combined with immunocytochemistry replicated the findings cited above. Crucially however, we added valuable spatial information on the location of *Dkk2^+^* microglia with respect to neurodegenerative lesions such as βAmyloid plaques in AD mouse models, where *Dkk2^+^* microglia exhibited a potential to cluster near βAmyloid plaques that was greater or at least equal to that of *Dkk2^–^* microglia, at least in advanced-*APP^NL-G-F^* mice. The exact role of Dkk2 protein expression is yet to be fully understood, but assuming its reported role as a secreted, soluble protein (for review, see Niehrs, 2006), we speculated that Dkk2’s mechanism of action could be autocrine or paracrine in nature. In support of the former, oncological evidence suggests that peripheral immune natural killer and CD8^+^ T cells, which are derived from the same myeloid lineage as CNS microglia, can detect soluble Dkk2. However, in this context, Dkk2 was utilized as an immune evasion tool secreted by tumors to suppress cytotoxic immune cell activation and tumor destruction via an atypical, Wnt signaling independent pathway (Xiao et al., 2018). Nonetheless, it is a possibility that microglial-derived Dkk2 can also act on microglia in an autocrine fashion at least in mice, although we can only speculate what the cellular response to such a stimulus would be.

Conversely, we provide evidence in support of a paracrine mechanism at least in cultured rat primary neurons as we demonstrate that recombinant human DKK2 protein blocks the synaptogenic effect of Wnt *in vitro*. However, we note that the administered DKK2 protein concentration may not match physiological, microglia derived Dkk2/DKK2 protein levels *in situ*. Knowing that DKK2 can generally engage in Wnt antagonizing and agonizing activities depending respectively on the presence or absence of the co-receptor Kremen2 (Mao and Niehrs, 2003), it appears that, at least in our *in vitro* system, DKK2 protein acts as an antagonist. DKK2 may thus behave similarly to DKK1, a negative regulator of canonical Wnt/β-catenin and noncanonical Wnt/PCP signaling with known synapse destabilizing properties (Purro et al., 2012; Galli et al., 2014; Killick et al., 2014; Marzo et al., 2016; Elliott et al., 2018; Sellers et al., 2018; also, for review, see Aghaizu et al., 2020), likely also in the human AD brain (Caricasole et al., 2004). Synapse density reductions in plaque proximal regions (Koffie et al., 2009) would be consistent with the fact that oligomeric βAmyloid induces *Dkk1* expression (Purro et al., 2012; Killick et al., 2014; Jackson et al., 2019). *Dkk2^+^* microglia accumulating around βAmyloid plaques may locally increase Dkk2 protein levels, adding to the anti-synaptic milieu established by Dkk1 near plaques. Given that microglia already engage in complement-mediated synaptic pruning by phagocytosis in AD mouse models (Hong et al., 2016; Shi et al., 2017), the relative contributions of individual synaptotoxic components around plaques will have to be addressed in future studies.

In assessing the chronological order between microgliosis/microglial plaque clustering and microglial *Dkk2* upregulation, we observed significant microgliosis increases before *Dkk2* upregulation in *APP/PS1*, *APP^NL-G-F^*, and *SOD1^G93A^* mice with respect to absolute quantification metrics (Figs. 1, 2, 4). However, when comparing relative rate changes, the rate of *Dkk2* signal increase at early disease stages in the *APP/PS1* AD mouse model surpassed the rate of microgliosis increase (Fig. 2*F*,*J*). It should be noted that *Dkk2* induction was initiated from near-zero basal expression levels (Fig. 2*E*), whereas both basal microglia numbers and Iba1 immunoreactivity levels were decidedly greater than zero (Fig. 2*C*,*D*,*G*,*H*). The potential for more pronounced changes was thus markedly greater for *Dkk2* induction at least in *APP/PS1* mice. Conversely, in S*OD1^G93A^* ALS mice, the rate of microgliosis increase surpassed that of *Dkk2* signal increase at early disease stages (Fig. 4*E*). Presumably, basal microglial cell densities lower than those observed in the mouse brain (Figs. 4*B*,*C* vs 2*C*,*D*,*G*,*H*), which is in keeping with published literature (Tan et al., 2020), likely contributed at least partially to this outcome.

What should be addressed in future studies is whether the ability to induce *Dkk2* expression is innate in all microglia or whether context, such as proximity to neurodegenerative lesions, is to be ascribed a more prominent role. CNS microglia are not a homogeneous population of cells, with gene expression signatures differing depending on factors such as brain region, sex, age, and context including disease (for review, see Masuda et al., 2020). In three-month-old *APP^NL-G-F^* mice, *Dkk2^+^* ARM cells represented 6% of to the total microglial pool (Sala Frigerio et al., 2019); this number increased to 33% and 52% at 6 and 12 months, respectively. It will be interesting to discern whether ARM-competence is restricted to the initial population of ARM cells, which then serve as a proliferating seed population, or whether cells from the total microglia pool are continuously recruited into the *Dkk2^+^* ARM population as disease progresses. The potential to produce *Dkk2^+^* ARM may further by influenced by other factors, which should be addressed in future studies, as different neurodegeneration disease models and CNS regions analyzed in our study exhibited varying contributions of *Dkk2^+^* microglia relative to the total microglial pool.

Finally, our study has revealed discrepancies between human AD and transgenic AD mouse models. *DKK2* mRNA expression levels were not elevated in postmortem frontal cortex samples from individuals diagnosed with AD versus healthy individuals. While other human brain and CNS regions like the motor cortex, hippocampus, and spinal cord might exhibit *DKK2* upregulation (although unlikely given the absence of *DKK2* upregulation in recently published human RNA-Seq databases; see below), the above finding is in stark contrast to our findings in neurodegeneration mouse models. In contrast to the situation in human patients, proximity to βAmyloid plaques appeared to be a strong predictor of microglial *Dkk2* expression in mice, both in the hippocampus and motor cortex. We note that those microglia that exhibited *DKK2* expression at the mRNA level in human tissue also displayed higher levels of the microglial marker *P2RY12*, but not *TREM2*. While the relevance of this finding is yet to be determined, published research has shown microglial expression of *P2RY12*, typically considered a homeostatic microglial marker gene, in proximity to diffuse plaques in postmortem tissue from AD individuals (Walker et al., 2020). A caveat worth mentioning in relation to the lack of *DKK2* upregulation is the fact that human microglia at AD end stage (Braak & Braak stage 5–6) were chronically exposed to disease for much longer periods than their mouse counterparts and chronic adaptations in microglia gene expression signatures as well as microglial numbers may have masked potential earlier changes (we note that our pathologic aging samples at Braak & Braak stage 3–4 also lacked *DKK2* upregulation). Nevertheless, it is now known that gene expression signatures between mouse and human DAM/ARM populations, although overlapping to some extent, exhibit distinct differences (for review, see Wang, 2021). In fact, numerous single-cell RNA-Seq analyses have identified gene expression signatures that differed between mouse and human DAM/ARM populations (Grubman et al., 2019; Mathys et al., 2019; Nguyen et al., 2020; Olah et al., 2020; Smith et al., 2022). For technical reasons and in contrast to mouse studies, human single-cell RNA-Seq studies are frequently, although not exclusively (Olah et al., 2020), restricted to nuclear transcripts, which may have contributed to the apparent transcriptomic differences between mouse and human microglia (note that extra-nuclear mRNA is abundant because of nuclear export before translation). However, even in a recent single nucleus RNA-Seq comparative study involving human AD postmortem tissue and the *5xFAD* AD mouse model, differences between human and mouse microglial gene expression signatures persisted (Zhou et al., 2020). The inability to detect extra-nuclear mRNA in human brain samples can be circumvented with the use of optimized tissue harvesting protocols (Olah et al., 2020), or *in situ* detection methods such as low throughput mRNA FISH (present study; Jolly et al., 2019) or higher throughput digital spatial profiling (Prokop et al., 2019). Nonetheless, our mRNA FISH based study strengthens the notion that human and mouse microglia, despite exhibiting some overlaps, are different even beyond just the expression status of *DKK2/Dkk2*, at least in the brain. Future studies should also examine any such interspecies differences in the spinal cord.

Our study therefore highlights the increasingly recognized difficulties and limitations of using mouse models to recapitulate facets of human biology and disease (Elder et al., 2010; Jucker, 2010; Cavanaugh et al., 2014; Justice and Dhillon, 2016; Perlman, 2016; Dawson et al., 2018). Regardless of whether this may be ascribed in our study to differing biological responses in humans versus mice or masking chronic adaptations in much longer human disease, these limitations likely play a key role in the absence of truly disease altering therapies to date despite decades of AD research and >100 clinical trials. Future AD research should thus substantially increase scrutiny in cases where animal models are to be used to ensure faithful modeling of human biology. Human based AD models including human induced pluripotent stem cell-derived cell cultures and brain organoids are potent additions to our tool-kit despite still lacking the capacity to fully recapitulate human *in vivo* biology in an *in vitro* setting, and indeed in an *in vivo* setting (Mancuso et al., 2019).

## Synthesis

Reviewing Editor: Francisca Bronfman, Universidad Andres Bello Facultad de Medicina Decisions are customarily a result of the Reviewing Editor and the peer reviewers coming together and discussing their recommendations until a consensus is reached. When revisions are invited, a fact-based synthesis statement explaining their decision and outlining what is needed to prepare a revision will be listed below. The following reviewer(s) agreed to reveal their identity: Ananya Chowdhury, Lorena Varella-Nallar.

The manuscript titled ‘Microglial expression of the Wnt signalling modulator DKK2 differs between human Alzheimer’s disease brains and mouse neurodegeneration models’ show an increase in microgliosis and microglial overexpression of Dkk2 mRNA (a homologue of Wnt-signaling antagonist- DKK1) in cortex and hippocampus of AD mouse models as well as the spinal cord of an ALS mouse model. An increase that correlates with the severity of the disease. The authors also show that the activated Dkk2+ microglia preferentially accumulated close to the βAmyloid plaques. However, the over-expression of Dkk in microglia was not detected in the human post-mortem tissues from patients with AD or pathological aging adding to the limitations of mouse models of AD to recapitulate the disease.

Reviewers agree that the manuscript is written clearly and the different experimental conditions have been explained properly with enough power and proper statistics to support their findings.

Nevertheless, some points need to be addressed before publication to strengthen the manuscript;

Major points:

1) On multiple occasions, the authors described their data without indicating clearly that they are referring to age-matched controls and not to data derived from the same mice at different time points. Particularly, the authors are comparing two separate groups of mice, thereby the values can not increase or decrease. Instead, they should indicate that those values are significantly higher or lower in disease mice compared to age-matched controls.

2) The methodology applied to randomly assign the plaque dense core “placeholders” for the conditions where no or minimal plaque was observed is not explained clearly in the paper. Please include this information in the methodology section.

3)In it is not clear in the RNA FISH analysis, why did the authors evaluate the signal area per microglial cell, which is very low. It is more appropriate to measure the number of puncta per cell, as well as the intensity and/or area of each spot.

4) It is not clear how the area f Dkk2 signal per cell was normalized.

5) The percentage of Iba1+ microglial cells expressing Dkk2 should be evaluated in Figures 1, 2, and 4. Also, it would be interesting to evaluate in human post-mortem brain samples the percentage of microglial cells expressing DKK2.

6) it is difficult to discriminate between magenta and red in Figures 6A-C, it seems that the density of cells expressing DKK2 is increased in Figures 6B-C. Thus, evaluating the percentage of microglial cells positive for DKK2 would be of interest. Also, potential differences in the expression of TREM2 and P2RY12 in DKK2+ cells should be analyzed.

7) Please indicate whether there are any morphological changes in cells expressing Dkk2? Or changes in the expression of Iba1 or any other DAM/ARM marker gene in Dkk2+ cells compared to Dkk2- cells?

8) How do the authors explain that no differences in the number of microglial cells between control, pathological aging and AD groups were observed in human samples? Was that expected? Please discuss.

9) Different regions were analyzed in mouse and human brain samples. Could this be relevant to the observed differences? This must be discussed mainly considering that, as mentioned in the discussion section, CNS microglia are not a homogeneous population.

Minor points:

1) Homologue has been used on pages 2 and 4 and homolog on page 26.

2) The second half of the schematic in Figure 3 A might have been cut off.

## Author Response

We would like to thank the Editor for accepting (in principle) this manuscript for publication.

We furthermore thank all the reviewers for their valued feedback and suggestions/recommendations. We have taken on board the suggestions/recommendations put forward by the Editor and are thus pleased to submit a revised manuscript that we feel to be well suited for this journal. Below, we briefly outline in blue text font how each of these suggestions/recommendations have been implemented in our manuscript.

Major points

1) On multiple occasions, the authors described their data without indicating clearly that they are referring to age-matched controls and not to data derived from the same mice at different time points. Particularly, the authors are comparing two separate groups of mice, thereby the values can not increase or decrease. Instead, they should indicate that those values are significantly higher or lower in disease mice compared to age-matched controls. We have amended relevant passages throughout the manuscript as suggested. We did not perform any longitudinal studies with our study subjects (mice/humans) and data are therefore always relative to (age-matched) controls.

2) The methodology applied to randomly assign the plaque dense core “placeholders” for the conditions where no or minimal plaque was observed is not explained clearly in the paper. Please include this information in the methodology section. We have now included this information in the methodology section as requested.

3) In it is not clear in the RNA FISH analysis, why did the authors evaluate the signal area per microglial cell, which is very low. It is more appropriate to measure the number of puncta per cell, as well as the intensity and/or area of each spot. We thank the reviewer(s) for their valuable feedback. We opted for the signal area per microglial cell metric rather than number of puncta per cell or puncta intensity due to the high propensity of microglia to cluster together close to amyloid plaques (see Fig. 3). Conceivably, in maximum projected confocal micrographs, considerable puncta undercounting caused by puncta overlap in 2D due to microglial clustering or puncta intensity miscalculations would have been evident. The signal area per microglial cell metric provided a more liberal, yet still highly accurate approach to quantifying the increase in Dkk2/DKK2 signal without becoming restricted by the limitations of having to define exact numbers of puncta. While our approach was still susceptible to underestimation error due to puncta overlap in 2D, this only strengthens our already highly significant results.

4) It is not clear how the area f Dkk2 signal per cell was normalized. We have now included this information in the methodology section as requested. Briefly, normalised Dkk2/DKK2 area per microglial cell was determined by dividing the total measured Dkk2/DKK2 area by the number of detected Iba1+ /DAPI+ (mouse) or DAPI+ /P2RY12+ /TREM2+ (human) microglia within a given field of view.

5) The percentage of Iba1+ microglial cells expressing Dkk2 should be evaluated in Figures 1, 2, and 4. Also, it would be interesting to evaluate in human post-mortem brain samples the percentage of microglial cells expressing DKK2. We have now included this quantification metric as requested. As part of these new quantifications, we have identified significant increases in the percentage of Dkk2+ /DKK2+ microglia in APPNL-G-F and SOD1G93A mice but not in APP/PS1 mice or brain biopsies from human individuals.

6) it is difficult to discriminate between magenta and red in Figures 6A-C, it seems that the density of cells expressing DKK2 is increased in Figures 6B-C. Thus, evaluating the percentage of microglial cells positive for DKK2 would be of interest. Also, potential differences in the expression of TREM2 and P2RY12 in DKK2+ cells should be analyzed. We have now rectified the colour clash in Figures 6A-C by replacing the magenta colour channel by yellow. As per 5), we have now quantified the percentage of DKK2+ microglial cells but found no significant differences between post-mortem brain tissues from healthy control, pathological ageing, and AD individuals. We have furthermore quantified TREM2 and P2RY12 expression levels at the mRNA level in DKK2+ and DKK2- microglial cell populations in post-mortem brain tissues from healthy control, pathological ageing, and AD individuals. While TREM2 levels were very similar in DKK2+ and DKK2- microglia, P2RY12 levels were higher in DKK2+ microglia compared to DKK2- microglia in healthy control, pathological ageing, and AD individuals, even though this difference was not statistically significant.

7) Please indicate whether there are any morphological changes in cells expressing Dkk2? Or changes in the expression of Iba1 or any other DAM/ARM marker gene in Dkk2+ cells compared to Dkk2- cells? We thank the reviewers for their valued feedback. As we have shown in Figure 3, most microglia in our AD mouse models exhibited a high degree of clustering around amyloid plaques at advanced disease stages. Thus, whereas morphological assessments of individual microglia would have been possible in littermate control mice or early disease stage AD mouse models, the clustering of microglia at advanced disease stages made it virtually impossible to assess the morphology of individual microglia cells in locations that appeared the most relevant (i.e., in close proximity to amyloid plaques). We used Iba1 IHC signal area as a proxy, but acknowledge that this approach lacks some of the detail that would have been generated through more sophisticated morphological analyses. We furthermore highlight that we have quantified the expression levels of microglial marker genes (TREM2, P2RY12) in DKK2+ and DKK2- microglia in human tissue (see point 6)).

8) How do the authors explain that no differences in the number of microglial cells between control, pathological aging and AD groups were observed in human samples? Was that expected? Please discuss. We have now addressed these questions as requested. The absence of microglia number changes in human individuals is in line with findings from published literature (e.g.: Marlatt et al., 2014; Davies et al., 2017; Paasila et al., 2019; Franco-Bocanegra et al., 2021). We can only speculate on the lack of microglial number differences between the different groups, but propose that this might have potentially been due to the long duration of the human disease condition where possible early changes in microglial numbers were overlooked by only investigating post-mortem tissue, in which disease caused changes had already occurred for 10+ years.

9) Different regions were analyzed in mouse and human brain samples. Could this be relevant to the observed differences? This must be discussed mainly considering that, as mentioned in the discussion section, CNS microglia are not a homogeneous population. We have highlighted the relevant section in the discussion, where we have addressed this issue. It is true that other human brain and CNS regions like the motor cortex, hippocampus, and spinal cord might exhibit DKK2 upregulation, but we consider this to be unlikely given the absence of DKK2 upregulation in recently published human RNA-seq databases, where some of these CNS regions were investigated (Grubman et al., 2019; Mathys et al., 2019; Nguyen et al., 2020; Olah et al., 2020; Smith et al., 2022).

Minor points:

1) Homologue has been used on pages 2 and 4 and homolog on page 26.

We have addressed this as requested and thank the reviewers for their feedback.

2) The second half of the schematic in Figure 3 A might have been cut off.

We thank the reviewers for their feedback. This was a stylistic choice to reflect the reality of acquiring confocal micrographs where the boundaries of the field of view frequently cut off imaged features at the edges of the field of view.
