# Supplementary material for: Microglial Expression of the Wnt Signaling Modulator DKK2 Differs between Human Alzheimer’s Disease Brains and Mouse Neurodegeneration Models
Source: eNeuro. 2023 Jan 9;10(1):ENEURO.0306-22.2022. doi: 10.1523/ENEURO.0306-22.2022 (PMC9836029; doi:10.1523/ENEURO.0306-22.2022)
Supplement: Extended Data Table 6-1 — Human sample demographic data. Related to Figure 6. Table listing demographic data of individual subjects contributing to the generation of dataset in Figure 6. Clinical presentation as well as postmortem brain assessments are shown [brain weight, postmortem (PM) delay, Braak & Braak stage, CERAD score, THAL stage, and ABC score]. Download Table 6-1, DOCX file. [file enu-eN-NWR-0306-22-s07.docx]

| Sample ID (FCx) | Sample classification | Age at onset | Age at death | Duration (yrs) | Gender | Clinical presentation | Brain Weight (g) | PM delay (hrs:min) | Braak and Braak | CERAD | THAL | ABC score |
| --- | --- | --- | --- | --- | --- | --- | --- | --- | --- | --- | --- | --- |
| P48/15 | Control |  | 84 |  | M | Normal | 1468 | 79:10 | 0 | none | 0 | A0 B0 C0 |
| P58/10 | Control |  | 87 |  | F | Normal | 1114 | 51:40 | 1 | mild | 1 | A1 B1 C1 |
| P66/11 | Control |  | 86 |  | F | Normal/ path ageing | 1234 | 120:00 | 2 | none | 0 | A0 B1 C0 |
| P64/11 | Control |  | 80 |  | F | Normal/ path ageing | 1242 | 49:10 | 2 | none | 0 | A0 B1 C0 |
| P94/05 | Control |  | 71 |  | M | Normal/ Mild cerebrovascular disease | 1480 | 38:50 | 1 | none | 0 | A0 B1 C0 |
| P61/09 | Pathological aging |  | 99 |  | F | Normal/ path ageing | 1141 | 32:05 | 4 | mod dif/ mod mature | 3 | A2 B2 C2 |
| P5/05 | Pathological aging |  | 85 |  | F | Pathological ageing | 1263 | 39:10 | 3 | Mod diff/ Sparse mature | 3 | A2 B2 C1 |
| P12/10 | AD | 44 | 56 | 12 | F | Amnestic | 1034 | 53:00 | 6 | frequent | 5 | A3 B3 C3 |
| P73/08 | AD | 50 | 61 | 11 | M | Amnestic | 1372 | 54:10 | 6 | frequent | 5 | A3 B3 C3 |
| P88/10 | AD | 50 | 66 | 16 | F | Amnestic | 906 | 92:47 | 6 | frequent | 5 | A3 B3 C3 |
| P40/10 | AD | 51 | 62 | 11 | F | Amnestic | 978 | 62:55 | 6 | frequent | 5 | A3 B3 C3 |
| P71/10 | AD | 65 | 70 | 5 | F | Amnestic | 1233 | 46:58 | 5 | moderate | 5 | A3 B3 C3 |
| P76/11 | AD | 65 | 72 | 7 | M | Amnestic | 1325 | 38:55 | 5 | moderate | 5 | A3 B3 C3 |
