# Supplementary material for: Microglial Expression of the Wnt Signaling Modulator DKK2 Differs between Human Alzheimer’s Disease Brains and Mouse Neurodegeneration Models
Source: eNeuro. 2023 Jan 9;10(1):ENEURO.0306-22.2022. doi: 10.1523/ENEURO.0306-22.2022 (PMC9836029; doi:10.1523/ENEURO.0306-22.2022)
Supplement: Extended Data Table 6-2 — mRNA FISH signal detection parameters. Related to Materials and Methods as well as Figure 6. Signal detection parameters used to identify DKK2, TREM2, and P2RY12 mRNA FISH signal on confocal images form human samples using HALO software with the FISH-IF v2.0.4 module (Indica Labs). Download Table 6-2, DOCX file. [file enu-eN-NWR-0306-22-s08.docx]

|  | *DKK2* | *P2RY12* | *TREM2* |
| --- | --- | --- | --- |
| Contrast threshold | 0 | 0 | 0.71 |
| Signal minimum intensity | 0.13 | 0.13 | 0 |
| Spot size | 0.5, 20 | 0.5, 20 | 0.5, 20 |
| Copy intensity | 0.15 | 0.15 | 0.17 |
| Segmentation aggressiveness | 0.95 | 0.95 | 0.318 |
