# Supplementary figures and images for: Microglial Expression of the Wnt Signaling Modulator DKK2 Differs between Human Alzheimer’s Disease Brains and Mouse Neurodegeneration Models
Source: eNeuro. 2023 Jan 9;10(1):ENEURO.0306-22.2022. doi: 10.1523/ENEURO.0306-22.2022 (PMC9836029; doi:10.1523/ENEURO.0306-22.2022)

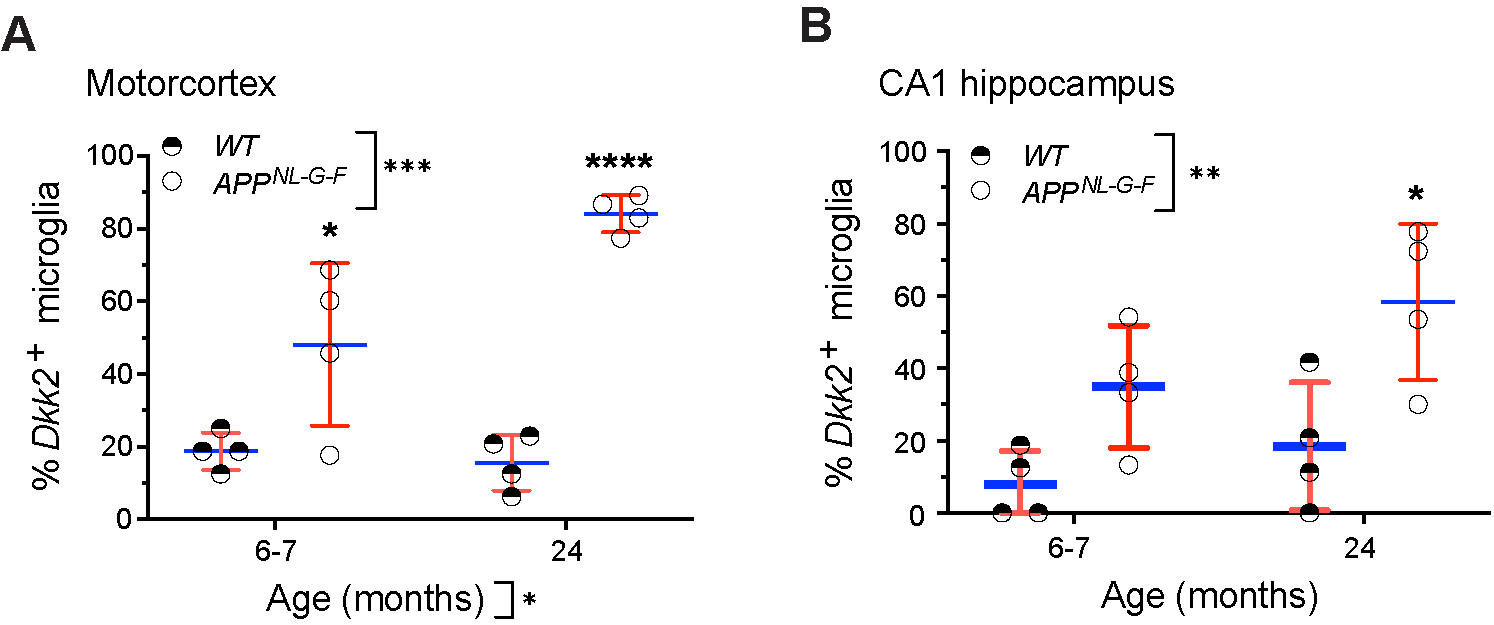

Supplement: Extended Data Figure 1-1 — Microglial Dkk2 upregulation in APPNL-G-F mice – % Dkk2+ microglia. Related to Figure 1. Relative contribution (%) of Dkk2+ microglia versus the total microglia population in the motor cortex (A) and CA1 hippocampus (B) of APPNL-G-F mice as assessed by Dkk2 mRNA FISH as well as microglial Iba1 IHC labelling. Individual data points represent the average of four FOVs analyzed for each animal. N = 4 animals per condition and time point, n = 4 different fields of view/animal and brain region. Two-way ANOVA with multiple comparisons test. *p < 0.05, **p < 0.01, ***p < 0.001, ****p < 0.0001 (g, h). Download Figure 1-1, TIF file. [file enu-eN-NWR-0306-22-s02.tif]

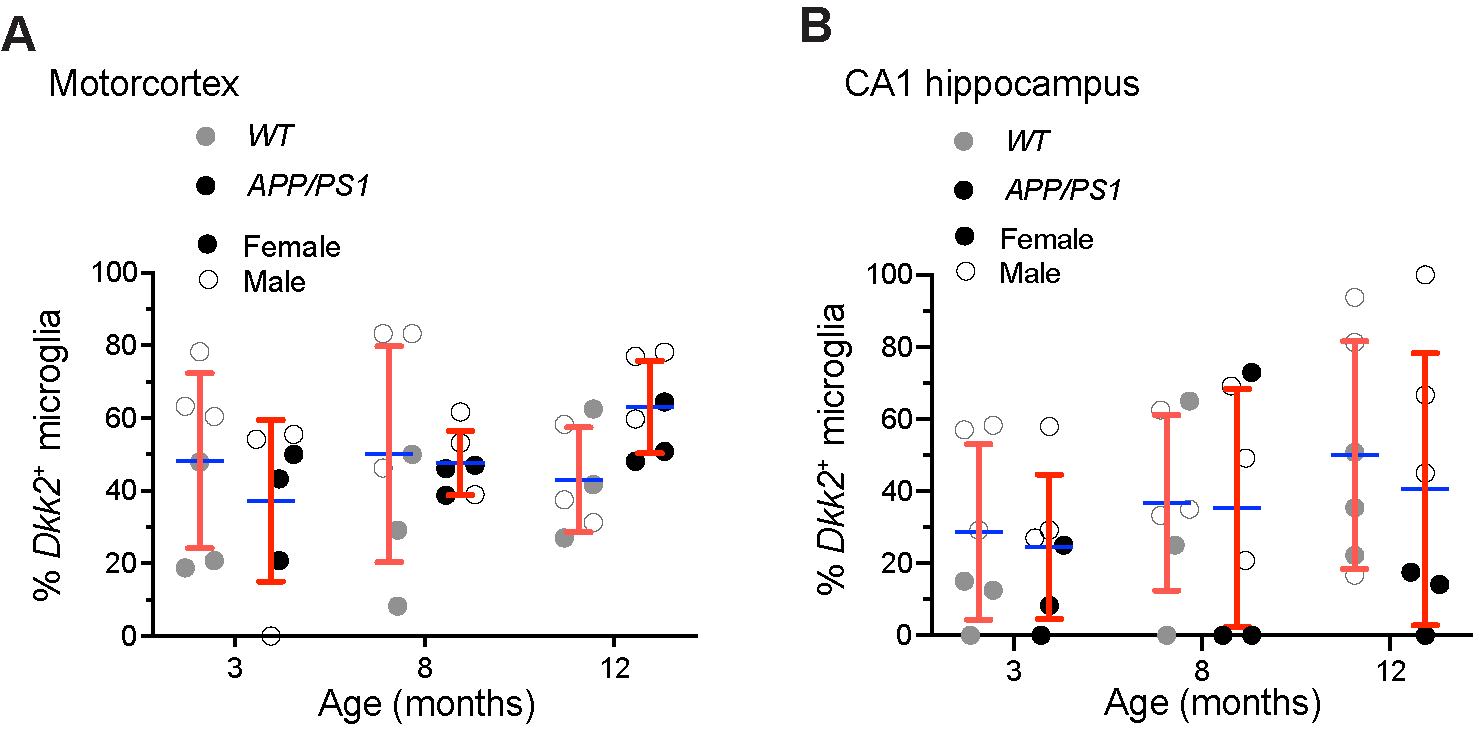

Supplement: Extended Data Figure 2-1 — Microglial Dkk2 upregulation in APP/PS1 mice – % Dkk2+ microglia. Related to Figure 2. Relative contribution (%) of Dkk2+ microglia versus the total microglia population in the motor cortex (A) and CA1 hippocampus (B) of APP/PS1 mice as assessed by Dkk2 mRNA FISH as well as microglial Iba1 IHC labelling. Individual data points represent the average of four FOVs analyzed for each animal. N = 6 animals (3× females, 3× males) per time point and condition, n = 4 different fields of view/animal and brain region. Two-way ANOVA with multiple comparisons test. *p < 0.05, **p < 0.01, ***p < 0.001, ****p < 0.0001 (l, m). Download Figure 2-1, TIF file. [file enu-eN-NWR-0306-22-s03.tif]

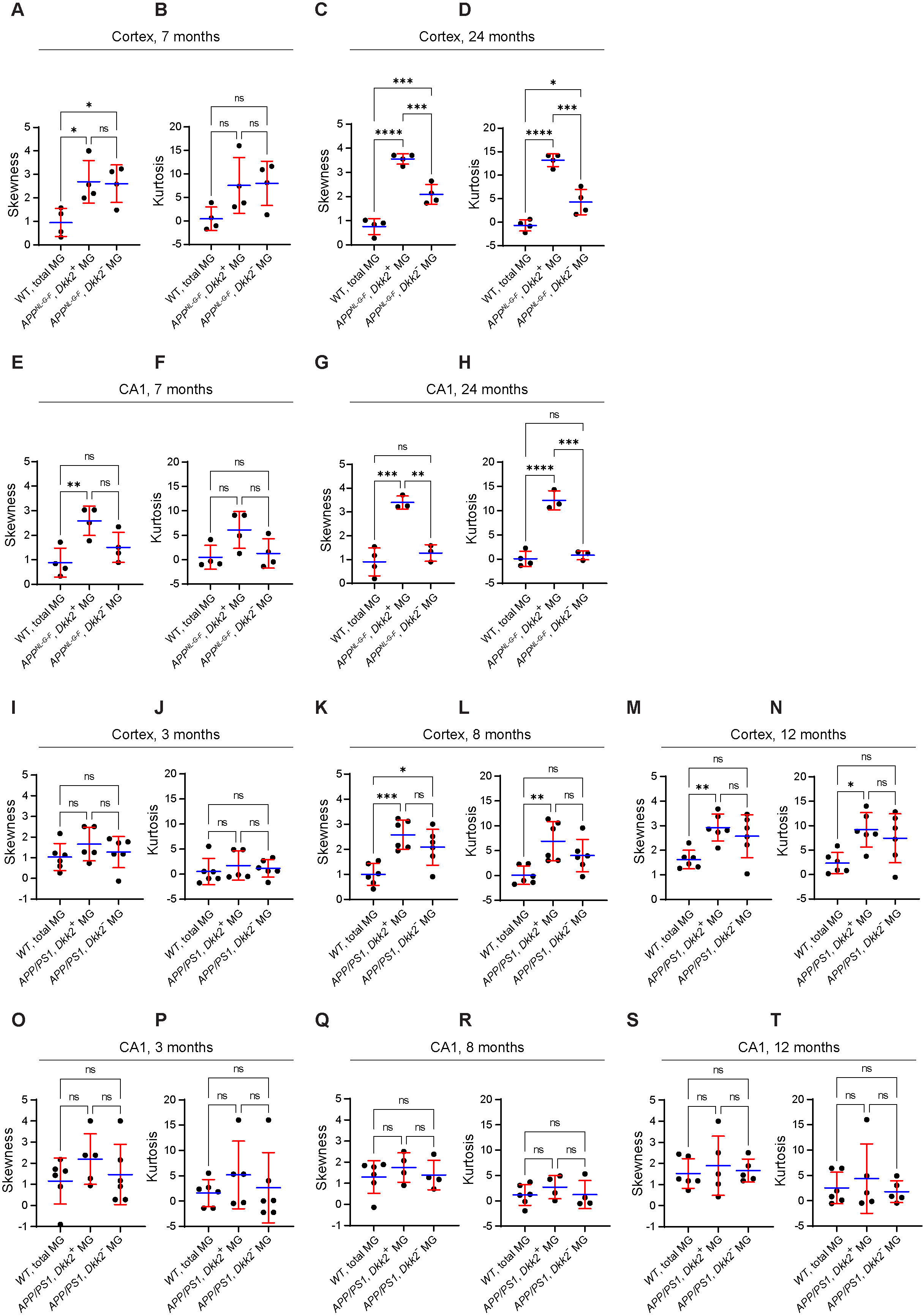

Supplement: Extended Data Figure 3-1 — Statistical analysis for microglial clustering around βAmyloid plaques. Related to Figure 3. A–H, Skewness and kurtosis analysis of histograms from microglia-βAmyloid plaque nearest neighbor analysis on APPNL-G-F mice in Figure 3 in the cortex (A–D and CA1 hippocampus (E–H) as well as at seven months (A, B, E, F) and at 24 months (C, D, G, H). I–T, Skewness and kurtosis analysis of histograms from microglia-βAmyloid plaque nearest neighbor analysis on APP/PS1 mice in Figure 3 in the cortex (I–N) and CA1 hippocampus (O–T) as well as at three months (I, J, O, P), eight months (K, L, Q, R), and at 12 months (M, N, S, T). Data points represent mean values for individual analyzed animals. APPNL-G-F/control: N = 4 animals per condition and time point, n = 4 different fields of view/animal and brain region; APP/PS1/control: N = 6 animals (3× females, 3× males) per time point and condition, n = 4 different fields of view/animal and brain region. One-way ANOVA with Tukey’s post hoc test. *p < 0.05, **p < 0.01, ***p < 0.001, ****p < 0.0001 (n–p). Download Figure 3-1, TIF file. [file enu-eN-NWR-0306-22-s04.tif]

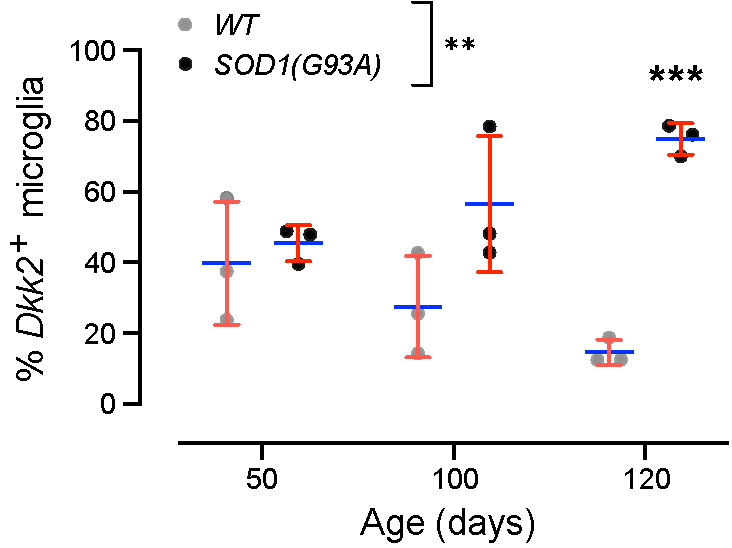

Supplement: Extended Data Figure 4-1 — Microglial Dkk2 upregulation in SOD1G93A ALS mice – % Dkk2+ microglia. Related to Figure 4. Relative contribution (%) of Dkk2+ microglia versus the total microglia population in the L5 ventral horn spinal cord of SOD1G93A ALS mice as assessed by Dkk2 mRNA FISH as well as microglial Iba1 IHC labelling. Individual data points represent the average of four FOVs analyzed for each animal. N = 3 animals per time point and condition, n = 4 fields of view per animal. Two-way ANOVA with multiple comparisons test. *p < 0.05, **p < 0.01, ***p < 0.001, ****p < 0.0001 (t). Download Figure 4-1, TIF file. [file enu-eN-NWR-0306-22-s05.tif]

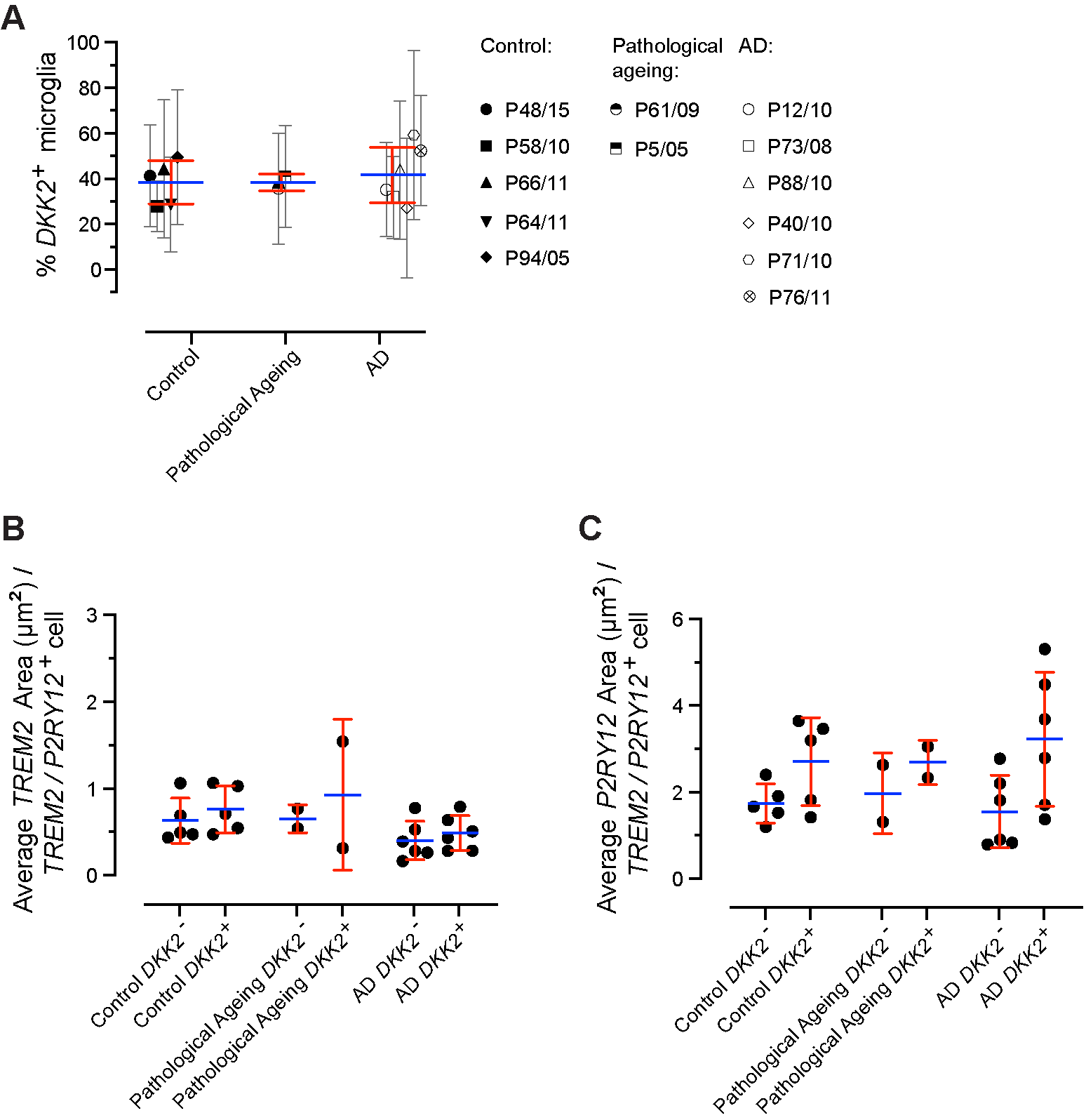

Supplement: Extended Data Figure 6-1 — DKK2, TREM2, and P2RY12 expression the mRNA level in human postmortem brains. Related to Figure 6. A, Relative contribution (%) of DKK2+ microglia versus the total microglia population in the human postmortem frontal cortex of control, pathological ageing, and AD individuals as assessed by DKK2, TREM2, and P2RY12 mRNA FISH. Normalized TREM2 (B) and P2RY12 (C) mRNA FISH signal area per DAPI+/TREM2+/P2RY12+ microglial cell in presence or absence of DKK2 expression. Healthy control individuals: N = 5 individuals, n = 8 fields of view); AD (Braak & Braak stage 5–6): N = 6 individuals, n = 8 fields of view; pathological ageing (Braak & Braak stage 3–4): N = 2 individuals, n = 8 fields of view. Data points represent the average of 8 FOVs analyzed for each individual subject (mean ± SD for A, mean for B, C); individual subject mean values were further averaged for each group of interest and summarized as mean ± SD (blue horizontal bars, red error bars). One-way ANOVA with Tukey’s post hoc test (y, z, ab). No statistical differences identified. Download Figure 6-1, TIF file. [file enu-eN-NWR-0306-22-s06.tif]
